# Bacterial Secretant from *Pseudomonas aeruginosa* Dampens Inflammasome Activation in a Quorum Sensing-Dependent Manner

**DOI:** 10.3389/fimmu.2017.00333

**Published:** 2017-03-27

**Authors:** Jungmin Yang, Kang-Mu Lee, Sangjun Park, Yoeseph Cho, Eunju Lee, Jong-Hwan Park, Ok Sarah Shin, Junghyun Son, Sang Sun Yoon, Je-Wook Yu

**Affiliations:** ^1^Department of Microbiology and Immunology, Institute for Immunology and Immunological Diseases, Brain Korea 21 PLUS Project for Medical Science, Yonsei University College of Medicine, Seoul, South Korea; ^2^Doping Control Center, Korea Institute of Science and Technology, Seoul, South Korea; ^3^Laboratory Animal Medicine, College of Veterinary Medicine and BK 21 PLUS Project Team, Chonnam National University, Gwangju, South Korea; ^4^Department of Biomedical Sciences, College of Medicine, Korea University Guro Hospital, Seoul, South Korea

**Keywords:** bacterial secretant, flagellin, inflammasome, *Pseudomonas aeruginosa*, quorum sensing

## Abstract

Inflammasome signaling can contribute to host innate immune defense against bacterial pathogens such as *Pseudomonas aeruginosa*. However, bacterial evasion of host inflammasome activation is still poorly elucidated. Quorum sensing (QS) is a bacterial communication mechanism that promotes coordinated adaptation by triggering expression of a wide range of genes. QS is thought to strongly contribute to the virulence of *P. aeruginosa*, but the molecular impact of bacterial QS on host inflammasome defense is completely unknown. Here, we present evidence that QS-related factors of the bacterial secretant (BS) from *P. aeruginosa* can dampen host inflammasome signaling in mouse bone marrow-derived macrophages. We found that BS from QS-defective Δ*lasR/rhlR* mutant, but not from wild-type (WT) *P. aeruginosa*, induces robust activation of the NLRC4 inflammasome. *P. aeruginosa*-released flagellin mediates this inflammasome activation by Δ*lasR/rhlR* secretant, but QS-regulated bacterial proteases in the WT BS impair extracellular flagellin to attenuate NLRC4 inflammasome activation. *P. aeruginosa*-secreted proteases also degrade inflammasome components in the extracellular space to inhibit the propagation of inflammasome-mediated responses. Furthermore, QS-regulated virulence factor pyocyanin and QS autoinducer 3-oxo-C12-homoserine lactone directly suppressed NLRC4- and even NLRP3-mediated inflammasome assembly and activation. Taken together, our data indicate that QS system of *P. aeruginosa* facilitates bacteria to evade host inflammasome-dependent sensing machinery.

## Introduction

The inflammasome, a cytoplasmic caspase-1-activating molecular complex, provides host with an innate immune defense against invading bacterial pathogens through induction of pyroptosis and production of pro-inflammatory cytokines such as interleukin-1 beta (IL-1β) and IL-18 (1). Multiple inflammasome sensor proteins are activated upon direct or indirect sensing of bacterial components in the cytoplasm (2). The cytosolic presence of bacterial flagellin or type 3 secretion system (T3SS)-associated proteins can promote the activation of the nucleotide-binding oligomerization domain-like receptor containing a caspase recruitment domain 4 (NLRC4) inflammasome with the engagement of neuronal apoptosis inhibitory protein, a direct sensor of bacterial proteins (3–5). In addition, bacterial double-stranded DNA (dsDNA) from phagocytosed bacteria is recognized in the cytoplasm by binding directly to absent in melanoma 2 (AIM2), which then assembles into the AIM2 inflammasome complex using dsDNA as a platform (6, 7). Furthermore, diverse bacterial toxins including pore-forming toxins (e.g., hemolysin, pneumolysin) and *Clostridium difficile* toxin B (TcdB) stimulate activation of NLRP3 and pyrin inflammasomes, respectively, by inducing intracellular alterations such as an efflux of potassium or the inactivation of Rho GTPases (8–10). Upon bacterial infections, these inflammasome sensor components assemble the inflammasome complex with ASC and procaspase-1 leading to caspase-1-dependent pyroptotic cell death and IL-1β or IL-18 secretion for efficient clearance of invading bacteria.

*Pseudomonas aeruginosa* is a Gram-negative, flagellated bacterium and an opportunistic pathogen (11). Immunocompromised hosts, such as cystic fibrosis patients, are likely to succumb to *P. aeruginosa* infection and suffer from chronic respiratory symptoms such as pneumonia (12, 13). This opportunistic bacterial pathogen produces multiple virulence factors involved in pathogenesis. Similar to other Gram-negative bacteria, *P. aeruginosa* uses T3SS to inject effector molecules such as exotoxins (e.g., ExoS, ExoU, ExoT, and ExoY) into the cytoplasm of host target cells (14). In this regard, the T3SS is required for *P. aeruginosa* virulence. Among the four previously mentioned exotoxins, ExoU is considered the most potent cytotoxin causing target cell death through its phospholipase activity (14). In addition to exotoxins, the flagella also contribute to successful *P. aeruginosa* pathogenesis through flagella-mediated bacterial motility and adhesion (15). Quorum sensing (QS), a form of bacterial cell-to-cell communication, also plays an important role in the pathogenicity of *P. aeruginosa* (16, 17). In the QS system of *P. aeruginosa, N*-acyl homoserine lactones (AHLs) such as *N*-3-oxo-dodecanoyl-l-homoserine lactone (3-oxo-C12-HSL) or *N*-butyryl-l-homoserine lactone (C4-HSL) are the most important communication signaling molecules and are referred to as autoinducer (18). Once the level of AHLs reaches a threshold because of increased bacterial communications, AHL binds to LasR or RhlR proteins. The resulting complex then acts as a transcription factor to induce the expression of many genes involved in the antibiotic production, biofilm formation, and virulence factors (18). However, the molecular impact of bacterial QS on the host innate immune response remains poorly described.

Innate immune defense against *P. aeruginosa* largely depends on the immediate recognition of essential bacterial virulence factors. In particular, monomeric flagellin, a basic component of flagella, is sensed by toll-like receptor 5 (TLR5) on the host cell surface leading to the production of pro-inflammatory cytokines (19). Furthermore, both flagellin and T3SS components such as PscC are required for *P. aeruginosa*-triggered activation of the NLRC4 inflammasome in a TLR5-independent manner (20, 21). Despite the critical role of bacterial QS in the virulence of *P. aeruginosa*, it remains poorly understood whether this bacterial communication signaling affects host inflammasome-mediated immune responses. Here, we explored a potential effect of QS in *P. aeruginosa* on inflammasome signaling and present molecular evidence that *P. aeruginosa* QS-related products could suppress the activation of inflammasome pathways.

## Materials and Methods

### Mice

C57BL/6 mice were purchased from Orient Bio. *Nlrp3^−/−^* mice were obtained from The Jackson Laboratory. *Nlrc4^−/−^* mice were kindly provided by Dr. G. Nunez (University of Michigan, Ann Arbor, MI, USA). All mice were on C57BL/6 background and 8–10 weeks male mice were used for the experiments. All mice were maintained under specific pathogen-free conditions.

### Ethics Statement

Protocols for the animal experiments were approved by the Institutional Ethical Committee, Yonsei University College of Medicine (2013-0164). All experiments were performed in accordance with the approved guidelines of the Institutional Ethical Committee, adhered to Guide for the Care and Use of Laboratory Animals of National Research Council (USA).

### Cell Cultures

Mouse primary bone marrow-derived macrophages (BMDMs) were differentiated from bone marrow cells isolated from mouse femurs. Immortalized wild-type (WT), *Asc^−/−^*, NLRP3-GFP-expressing BMDMs and ASC-GFP-expressing THP-1 cells were kindly provided by Dr. E. S. Alnemri (Thomas Jefferson University, Philadelphia, PA, USA). All BMDMs were maintained in L929 supplements-conditioned Dulbecco’s modified Eagle’s medium (DMEM) supplemented with 10% fetal bovine serum (FBS) and antibiotics. THP-1-ASC-GFP cells were grown in RPMI-1640 supplemented with 10% FBS, 2 mM glutamine, 10 mM HEPES, 1 mM sodium pyruvate, and 0.05 mM 2-mercaptoethanol and antibiotics. THP-1-ASC-GFP cells were differentiated into macrophage-like cells using a phorbol 12-myristate 13-acetate treatment and then used for experiments. A549 cells were cultured in DMEM supplemented with 10% FBS and antibiotics. Cell death was determined by the extracellular release of lactate dehydrogenase using a CytoTox96 non-radioactive cytotoxicity assay kit (Promega).

### Bacterial Cultures

*Pseudomonas aeruginosa* strain PAO1 was used. PAO1 deletion mutants *(ΔlasR/rhlR, ΔlasR, ΔrhlR, ΔfliC, ΔlasR/rhlR/fliC, ΔlasR/fliC*, and *ΔlasB*) were created by allelic replacement as previously described (22). For bacterial infection, all PAO1 strains were grown overnight in Luria-Bertani (LB) medium at 37°C with aeration, then diluted 1:50 with fresh LB medium, and grown for an additional 2 h. Bacteria were then pelleted by centrifugation and resuspended in Opti-MEM medium. The bacterial suspension was then added to the culture medium of BMDMs at the indicated multiplicity of infection. To prepare bacterial lysates, cultured PAO1 were lysed, sonicated, and centrifuged to remove insoluble pellets. The supernatants were normalized and used for immunoblotting.

### Bacterial Secretants (BSs)

For preparation of BS, PAO1 strains were grown overnight in LB medium and then diluted with fresh LB medium and grown for an additional 6 h. The bacterial culture was then normalized by the number of bacteria. The normalized bacterial suspension was centrifuged and filtered to remove bacteria, and the final filtrate was used as BS. For heat treatment, BSs were brought to heat at 98°C for 30 min to inactivate bacterial proteases activity. For protease treatment, BSs were incubated with proteinase K (100 μg/ml) at 37°C for 1 h and subsequently boiled at 98°C for 30 min to eliminate the proteinase K activity.

### Reagents and Antibodies

Flagellin purified from *P. aeruginosa* was purchased from InvivoGen. DOTAP liposomal transfection reagent was purchased from Sigma-Aldrich. Lipopolysaccharide (LPS), ATP, nigericin, pyocyanin, and proteinase K were purchased from Sigma-Aldrich. *N*-3-oxo-dodecanoyl-l-homoserine lactone (3-oxo-C12-HSL) and *N*-butyryl-l-homoserine lactone (C4-HSL) were obtained from Cayman. Antibodies were acquired to detect mouse caspase-1 (Adipogen, 20B-0042), mouse IL-1β (R&D, AF-401-NA), mouse IL-6 (Cell Signaling, 12912), mouse NLRP3 (Adipogen, 20B-0014), mouse ASC (Santa Cruz, SC-22514-R), and flagellin (InvivoGen, mabg-flapa).

### Immunoblot Analysis

Cells were harvested and then lysed in 20mM HEPES (pH 7.5) buffer containing 0.5% Non-idet P-40, 50mM KCl, 150mM NaCl, 1.5mM MgCl_2_, 1mM EGTA, and protease inhibitors. After centrifugation to remove cell debris, soluble lysates were fractionated by SDS-polyacrylamide gel electrophoresis, transferred to PVDF membranes (Bio-Rad), and then immunoblotted by western blotting. For some experiments, BMDM culture supernatants were collected after inflammasome stimulations, and proteins were precipitated by the addition of a methanol/chloroform mixture as reported previously (23), and then immunoblotted. All blots shown are representative images of at least three independent experiments. Images have been cropped for presentation.

### Determination of Inflammasome Activation

To trigger classical NLRP3 inflammasome activation, BMDMs were primed with LPS (0.25 μg/ml, 3 h) and then treated with ATP (2 mM, 45 min) or nigericin (5 μM, 45 min). To induce AIM2 inflammasome activation, BMDMs were transfected with poly dA:dT using lipofectamine according to the manufacturer’s protocol. Inflammasome activation was then determined by the detection of active caspase-1 p20 and active IL-1β in culture supernatants using immunoblots, and by quantification of extracellular IL-1β using the mouse IL-1β Quantikine ELISA kit (R&D systems). The levels of IL-6 in culture supernatants were quantified by mouse IL-6 Quantikine ELISA kit (R&D systems). For some experiments, BMDM supernatants were incubated with BS, subjected to protein precipitation by methanol/chloroform, and then immunoblotted. To deliver flagellin into the cytosol of BMDMs, flagellin (0.35 μg) was incubated with DOTAP liposomal transfection reagent (3.5 μl) in serum-free Opti-MEM medium (50 μl) at 25°C for 30 min. Then, 300 μl Opti-MEM medium was added, and the final mixture (250 μl total) was used to transfect BMDMs in a six-well plate.

### Determination of ASC Oligomerization

To detect ASC oligomerization, a chemical cross-linking assay was performed as described previously (6). Briefly, cells were lysed and centrifuged at 6,000 rpm for 10 min. The pellets were washed and resuspended in phosphate-buffered saline. The resuspended pellets were cross-linked with 1 mM discuccinimidyl suberate [disuccinimidyl suberate (DSS); Pierce, Rockford, IL, USA] for 30 min. The cross-linked pellets and soluble lysates were fractionated by SDS-PAGE and immunoblotted with anti-ASC antibody. To visualize ASC speck-like aggregates, THP-1 cells stably expressing ASC-GFP were observed by confocal microscopy.

### Quantification of mRNA

To measure mRNA levels, quantitative real-time PCR was performed. Briefly, total cellular RNA was isolated using the TRIzol reagent (Invitrogen) and reverse transcribed using PrimeScript RT Master Mix (Takara) according to the manufacturer’s instructions. Template DNA was amplified by quantitative real-time PCR using SYBR Premix Ex TaqII (Takara). Primers were as follows: 5′-GCC CAT CCT CTG TGA CTC AT-3′ and 5′-AGG CCA CAG GTA TTT TGT CG-3′ (*Il-1b*); 5′-AGT TGC CTT CTT GGG ACT GA-3′ and 5′-TCC ACG ATT TCC CAG AGA AC-3′ (*Il-6*); and 5′-CGC GGT TCT ATT TTG TTG GT-3′ and 5′-AGT CGG CAT CGT TTA TGG TC-3′ (*Rn18s*).

### Sample Preparation for Metabolomic Analysis

Selected solvents with different polarities (hexane, ethyl acetate, and acetonitrile) were used to extract metabolites from the samples. Each solvent was added to the same volume of sample, and the mixture was vortexed for 1 min. Samples were centrifuged (2,500 rpm, 5 min), the supernatant was removed and centrifuged again (15,000 rpm, 5 min), and 200 μl of the final supernatant was pipetted into sample vials with inserts.

### Ultra-Fast Liquid Chromatography (UFLC) Condition

The UFLC system was equipped with a DGU-20A5R degasser, LC-20AD XR binary pump system, SIL-20AC XR autosampler at 15°C, and CTO-20AC column oven at 35°C (Shimadzu). Chromatographic separation was conducted on a reversed-phase column (Imtakt Scherzo S-C18 column, 100 mm × 2.0 mm, i.d., 3 μm) at flow rate of 0.3 ml min^−1^. The mobile phases used were (A) water and (B) methanol, both containing 0.1% formic acid. The gradient program was set as follows: 98% (A) and 2% (B), held for 1 min; linear increase from 2 to 95% (B) over 14 min, held for 3 min; return to initial conditions in 0.01 min and equilibration for 1.99 min.

### High-Resolution Mass Spectrometry (HRMS)

High-resolution mass spectrometry detection was performed on both Exactive Plus Orbitrap and Q-Exactive Plus Quadrupole-Orbitrap mass spectrometers (Thermo) equipped with an electrospray ionization source and operated in positive mode. Screening and confirmation of the substances were performed using full scan mode in Exactive Plus and parallel reaction monitoring (PRM) mode in Q-Exactive Plus. For identification of selected molecules, the scan range and collision energy were applied to each substance at a resolution of 35,000 in PRM mode.

### UFLC–HRMS Data Processing

The raw data acquired by Xcalibur software (Thermo) were processed using MZmine2 software (24). After peak picking, mass detection, chromatogram building, and deconvolution, peaks were grouped depending on *m*/*z* and retention time value by RANSAC alignment and peak finder. The expected structure of candidates based on isotope rate and MS/MS (PRM mode) were identified by entering the formula and fragment data into mzCloud,^1^ MetFrag,^2^ and PubChem.^3^ The results for the most suitable candidates were then compared to standards by MS and MS/MS spectra.

### Statistical Analysis

All values were expressed as the mean and SE of three independent experiments unless otherwise indicated. Data were analyzed using one-way analysis of variance followed by Bonferroni *post hoc* test after checking the assumptions of normal distribution by Shapiro test and equal variance by Bartlett or Brown–Forsythe test. Otherwise, Welch’s *t*-tests were used for unequal variances. The *p* values ≤0.05 were considered significant. Statistical analyses were carried out using GraphPad Prism and R software.

## Results

### Prolonged *P. aeruginosa* Infection Impairs Inflammasome-Associated Molecules

**Figure 1:**
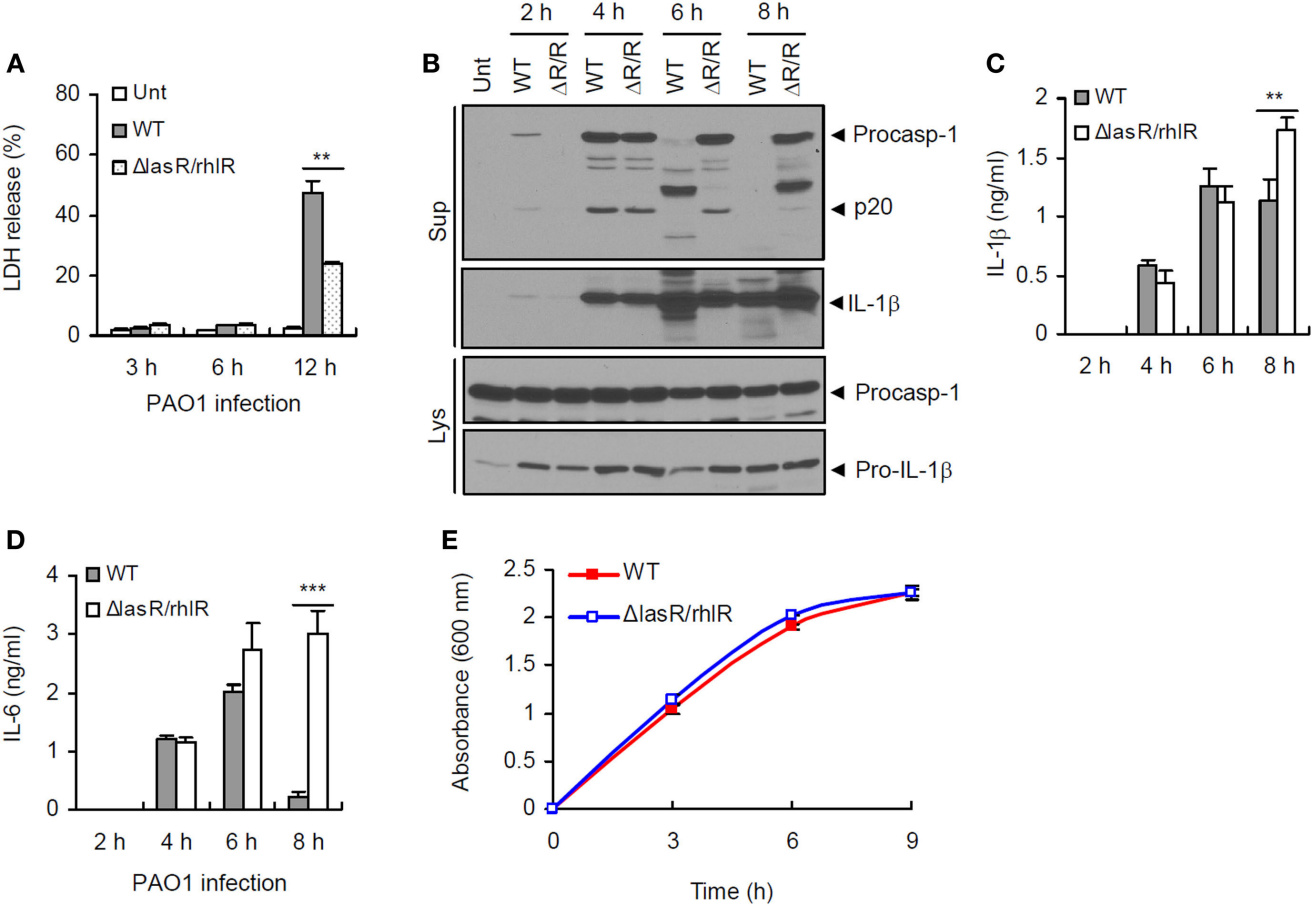
**Quorum sensing-dependent impairment of inflammasome signaling by prolonged *Pseudomonas aeruginosa* infection**. **(A)** Human lung epithelial A549 cells were infected with wild-type (WT) or *ΔlasR/rhlR P. aeruginosa* PAO1 at a multiplicity of infection (MOI) 3. At 3–12 h postinfection, A549 cell death was determined by lactate dehydrogenase (LDH) assay. Asterisk indicates significant difference between WT and *ΔlasR/rhlR* (*n* = 3). **(B)** Mouse bone marrow-derived macrophages (BMDMs) were untreated (Unt) or infected with WT or *ΔlasR/rhlR* (ΔR/R) *P. aeruginosa* PAO1 at MOI 3. At 2–8 h postinfection, culture supernatants (Sup) or cellular lysates (Lys) were immunoblotted with the indicated antibodies. **(C,D)** Mouse BMDMs were infected with WT or *ΔlasR/rhlR* PAO1 at MOI 2. Extracellular levels of interleukin-1 beta (IL-1β) **(C)** or IL-6 **(D)** were quantified by ELISA in the culture supernatants of BMDMs at the indicated time postinfection. Asterisks indicate significant difference between WT and *ΔlasR/rhlR* (*n* = 6). **(E)** Bacterial growth curve of WT or *ΔlasR/rhlR P. aeruginosa*. Statistical significance was determined by one-way analysis of variance with a Bonferroni post-test or Welch’s *t*-test (**P < 0.01; ***P < 0.001).

To examine a potential role of *P. aeruginosa* QS in host inflammasome activation, we prepared a QS-defective *ΔlasR/rhlR* double mutant strain of *P. aeruginosa* PAO1 bacteria lacking LasR and RhlR, two principal transcriptional regulators of *P. aeruginosa* QS system. At 12 h postinfection, *ΔlasR/rhlR* mutant showed a reduced cytotoxicity to human lung epithelial A549 cells compared with WT *P. aeruginosa* (Figure 1A), suggesting that QS-deficient *ΔlasR/rhlR* mutant is less virulent. Then, we assessed inflammasome activation in BMDMs upon infection with WT and QS mutant *P. aeruginosa*. At 4 h postinfection, both WT and *ΔlasR/rhlR* mutant strain promoted robust inflammasome activation as determined by the secretion of active caspase-1 (p20) and IL-1β into BMDMs culture supernatant (Figure 1B). Of notice, prolonged infection with WT *P. aeruginosa* (6 or 8 h), but not with *ΔlasR/rhlR*, caused a manifest degradation of caspase-1 and IL-1β in the culture supernatants (Figure 1B). Consistently, levels of secreted pro-inflammatory cytokines IL-1β and IL-6 at 8 h postinfection were significantly lower in BMDMs infected with WT *P. aeruginosa* than in those with the *ΔlasR/rhlR* mutant (Figures 1C,D). This apparent QS-mediated impairment of inflammasome signaling is not likely a consequence of reduced growth of the WT strain, because the growth kinetics of WT and QS-defective strains were similar (Figure 1E). These observations indicate that prolonged infection with *P. aeruginosa* could impair host inflammasome-mediated responses in a QS-dependent manner.

### *P. aeruginosa* Secretant Degrades Host Inflammasome Components in a QS-Dependent Manner

**Figure 2:**
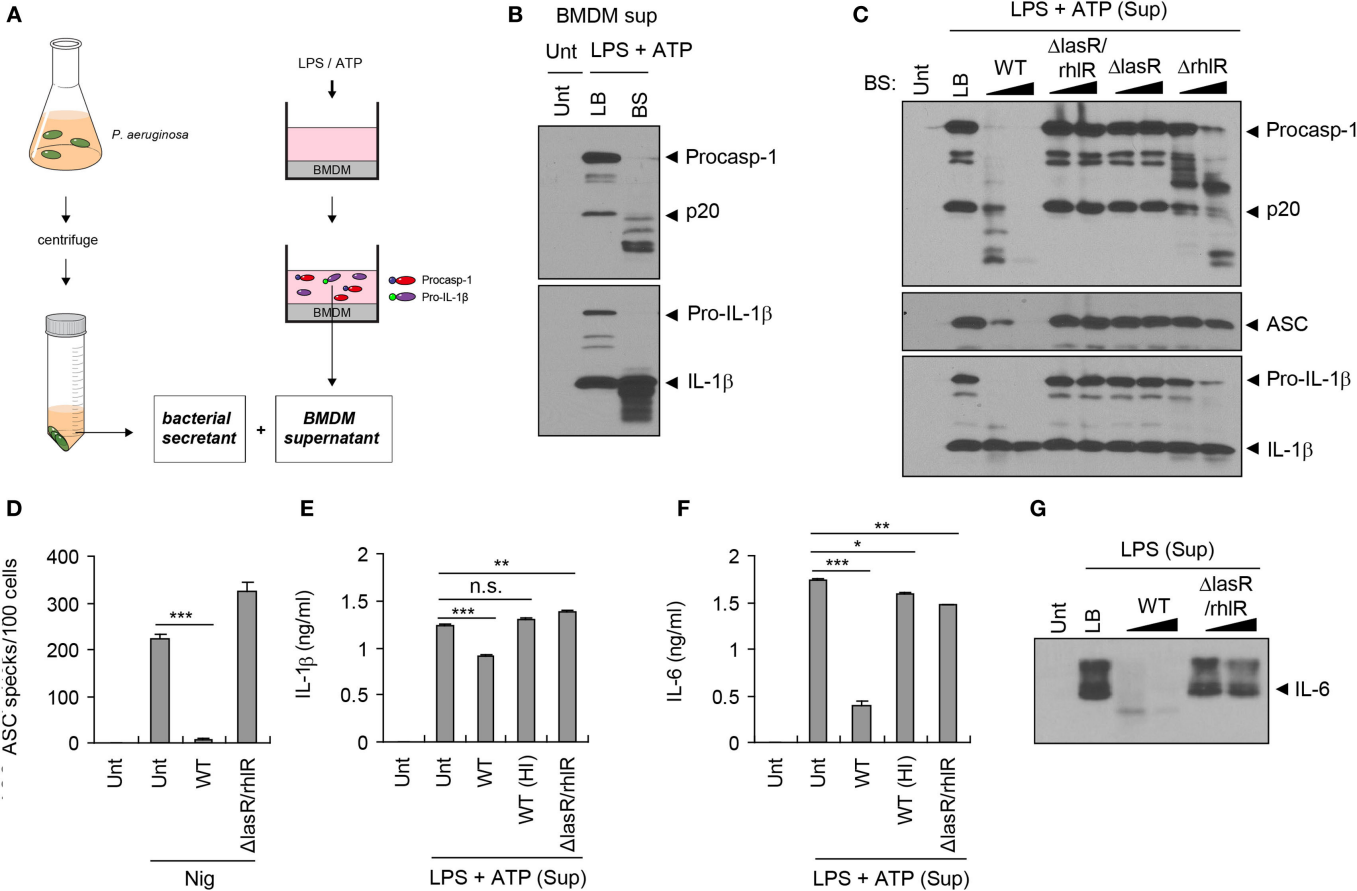
**Quorum sensing-dependent degradation of inflammasome components by *Pseudomonas aeruginosa* secretant**. **(A)** Illustration of experimental scheme showing a collection of bacterial secretant (BS) and bone marrow-derived macrophages (BMDMs) supernatant containing inflammasome components. BMDM culture supernatant was incubated with *P. aeruginosa* BS (1/10 volume of BMDM supernatant) at 37°C for 10–120 min. **(B)** Untreated or lipopolysaccharide (LPS)/ATP-stimulated BMDM culture supernatants were incubated with Luria-Bertani (LB) medium or PAO1 BS for 45 min. The mixtures were then precipitated and immunoblotted with the indicated antibodies. **(C)** LPS/ATP-stimulated BMDM culture supernatants were incubated with wild-type (WT), *ΔlasR/rhlR, ΔlasR*, or *ΔrhlR* secretant for 10 or 60 min, and immunoblotted with the indicated antibodies. **(D)** THP-1-ASC-GFP cells were treated with nigericin (5 μM, 3 h) in the presence or absence of WT or *ΔlasR/rhlR* secretant. The number of ASC specks was counted and displayed as specks per 100 cells. Asterisk indicates significant difference (*n* = 4). **(E,F)** LPS/ATP-stimulated BMDM culture supernatants were incubated with intact or heat-inactivated WT or Δ*lasR/rhlR* secretant at 37°C for 30 min. The levels of interleukin-1 beta (IL-1β) **(E)** or IL-6 **(F)** in the mixture were quantified by ELISA. Asterisks indicate significant differences (*n* = 3). HI, heat inactivation. **(G)** Untreated or LPS-treated (0.5 μg/ml, 5 h) BMDM culture supernatants were incubated with WT or *ΔlasR/rhlR* secretant for 10, 60 min, and then immunoblotted with anti-IL-6 antibody. Statistical significance was determined by one-way analysis of variance with a Bonferroni post-test (n.s., not significant; *P < 0.05; **P < 0.01; ***P < 0.001).

To test whether bacterial secreted factors are responsible for the impairment of host inflammasome-associated molecules shown above, we collected bacteria-free supernatant (referred to as bacterial secretant (BS)) from *P. aeruginosa* cultures (Figure 2A). Simultaneously, we collected culture supernatant from BMDMs upon stimulation with NLRP3 inflammasome-activating LPS/ATP, and this LPS/ATP-stimulated BMDM supernatant was then incubated with BS from the WT *P. aeruginosa* culture (Figure 2A). Both procaspase-1 and active caspase-1 p20 in the BMDMs supernatant were clearly processed into fragments by BS, as was the IL-1β secreted from BMDMs (Figure 2B).

*Pseudomonas aeruginosa* produces QS-regulated proteases such as elastase (LasB) and alkaline protease (AprA) to degrade host defense proteins such as extracellular matrix (ECM) components and cytokines (11, 25). To examine whether *P. aeruginosa* proteases are involved in the degradation of inflammasome-associated molecules, BS was heated to eliminate protease activity. Consequently, heat inactivation of BS markedly abolished the degradation of inflammasome components such as caspase-1 and ASC that was observed after incubation with intact *P. aeruginosa* secretant (Figure S1A in Supplementary Material). Furthermore, the secretant of the *ΔlasB* strain, lacking the major *P. aeruginosa* QS-dependent protease LasB, failed to efficiently degrade inflammasome components (Figure S1B in Supplementary Material), indicating that LasB plays a role in this degradation.

LasR is an essential transcriptional regulator in *P. aeruginosa* that induces the expression of many QS-related genes including proteases (18). Unlike WT *P. aeruginosa* secretant, neither *ΔlasR/rhlR* nor *ΔlasR* secretant was able to process inflammasome components (Figure 2C), confirming that the QS system is necessary for the degradation of host inflammasome components by BS. Although both LasR and RhlR are the principal transcriptional regulators of *P. aeruginosa* QS, LasR is a prerequisite regulator for the expression of RhlR (26). Partial processing of caspase-1 was detected in *ΔrhlR* mutant secretant (Figure 2C), suggesting that the secretant contains a minimal base level of proteases. Indeed, previous studies showed that *ΔrhlR* mutant produces a kind of proteases including AprA (27, 28).

Caspase-1 and IL-1β secreted from *P. aeruginosa*-infected BMDMs were also degraded by WT *P. aeruginosa* secretant, but not by heat-inactivated or *ΔlasR/rhlR* secretant (Figures S1C,D in Supplementary Material). Along with caspase-1 and IL-1β, aggregates of inflammasome adaptor protein ASC are secreted from BMDMs upon inflammasome activation, which could propagate the inflammatory responses (29, 30). Monomeric ASC in the LPS/ATP-stimulated supernatant was also degraded by WT BS, but not by *ΔlasR/rhlR* secretant (Figure 2C). In ASC-GFP-expressing THP-1 cells, WT *P. aeruginosa* secretant clearly reduced nigericin-triggered formation of ASC aggregates, which appear as specks by confocal microscopy, while *ΔlasR/rhlR* secretant failed to decrease the number of ASC specks (Figure 2D; Figure S1E in Supplementary Material). Consistently, the level of secreted IL-1β from LPS/ATP-treated BMDMs was significantly impaired by the incubation with WT secretant, but not with heat-inactivated nor *ΔlasR/rhlR* secretant (Figure 2E). Pro-inflammatory cytokine IL-6 secreted by BMDMs was also degraded by incubation with WT secretant, but less with QS mutant secretant (Figures 2F,G). All of these findings clearly demonstrate that BS from *P. aeruginosa* can degrade host inflammasome components and cytokines, enabling QS-dependent proteases to disrupt innate immune defenses.

### BS from QS Mutant, but Not from WT *P. aeruginosa*, Promotes Inflammasome Activation

**Figure 3:**
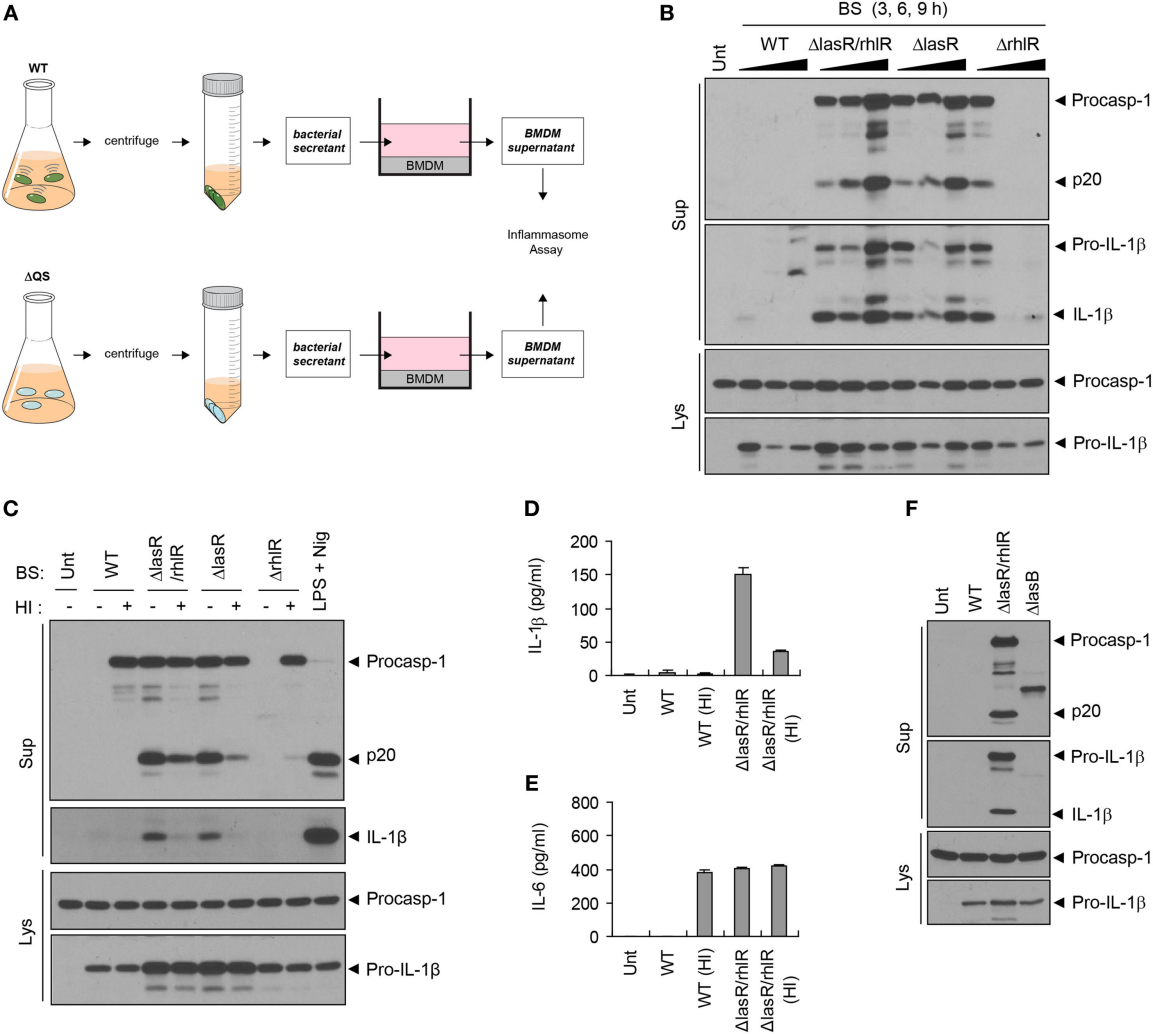
**Quorum sensing (QS)-defective *Pseudomonas aeruginosa* secretant triggers inflammasome activation**. **(A)** Illustration of experimental scheme to determine a potent role of bacterial secretant (BS) in inflammasome activation. BSs were prepared from wild-type (WT) or QS-defective mutant (ΔQS) *P. aeruginosa* cultures. BSs were then added to bone marrow-derived macrophage (BMDM) culture (at a 1:10 ratio) for 6 h, and BMDM culture supernatants were assayed for the inflammasome activity. **(B)** Mouse BMDMs were left untreated (Unt) or treated with BSs from WT, *ΔlasR/rhlR, ΔlasR*, or *ΔrhlR P. aeruginosa* 3-, 6-, or 9-h precultures. **(C)** Mouse BMDMs were treated with intact or heat-inactivated WT, *ΔlasR/rhlR, ΔlasR*, or *ΔrhlR* secretants for 6 h, or primed with lipopolysaccharide (0.25 μg/ml, 3 h), followed by treatment with nigericin (Nig, 5 μM, 45 min). **(D,E)** Mouse BMDMs were treated with intact or heat-inactivated WT or *ΔlasR/rhlR* secretants for 6 h. Cytokine levels of interleukin-1 beta (IL-1β) **(D)** or IL-6 **(E)** in the culture supernatants were quantified by ELISA (*n* = 3). HI, heat inactivation. **(F)** Mouse BMDMs were treated with WT, *ΔlasR/rhlR*, and *ΔlasB* secretants for 6 h. **(B,C,F)** Culture supernatants (Sup) or cellular lysates (Lys) were immunoblotted with the indicated antibodies.

Given the host protein-processing effect of the *P. aeruginosa* QS system, we further examined a potential role of BS, which contains diverse QS-dependent virulence factors, in host inflammasome signaling pathways. Thus, we treated BMDMs with bacteria-free WT or QS-defective *P. aeruginosa* culture secretants (Figure 3A). To our surprise, BS from *ΔlasR/rhlR* or *ΔlasR* mutant strains promoted robust caspase-1 activation and IL-1β secretion, while WT secretant failed to trigger caspase-1 activation (Figure 3B). Meanwhile, *ΔrhlR* secretant isolated from 6- or 9-h preculture did not cause a robust caspase-1 processing. Because *ΔrhlR* mutant seems to still secrete some proteases, the deficiency of caspase-1 and IL-1β in the supernatant of BMDMs treated with *ΔrhlR* secretant could be explained by bacterial protease-mediated degradation.

Consistent with caspase-1 activation, *ΔlasR/rhlR* mutant secretant, but not that of WT bacteria, caused a robust production of IL-1β and IL-6 by BMDMs (Figures S2A,B in Supplementary Material), while secretants from both WT and *ΔlasR/rhlR P. aeruginosa* induced upregulation of *Il-1*β and *Il-6* mRNA (Figures S2C,D in Supplementary Material). As for IL-6, it is likely that WT *P. aeruginosa* secretant could degrade IL-6 secreted from BMDMs *via* its proteases, because WT *P. aeruginosa* secretant triggered mRNA production of IL-6, subsequently leading to its secretion. On the other hand, the absence of caspase-1 and IL-1β in the supernatant of BMDMs treated with WT secretant could be explained by two possible scenarios. First, it is plausible that WT secretant may be unable to trigger inflammasome activation. The second hypothesis is that WT secretant may promote inflammasome activation, leading to the secretion of inflammasome proteins, but bacterial secreted proteases could then degrade caspase-1 and IL-1β in the extracellular supernatant. To test these possibilities, we performed similar experiments with heat-inactivated BS to avoid the involvement of proteases. As a result, WT secretant failed to induce caspase-1 activation and IL-1β secretion even after heat inactivation (Figures 3C,D). Instead, heat inactivation resulted in the appearance of procaspase-1 by WT secretant (Figure 3C), suggesting that secreted procaspase-1 may be degraded by bacterial proteases. These data demonstrate that WT secretant is unable to promote inflammasome activation regardless of protease activity. However, heat-treated WT secretant caused robust secretion of IL-6 (Figure 3E), suggesting that proteases from the WT secretant had been responsible for processing IL-6 in the extracellular space. To further clarify the involvement of proteases in the failure of inflammasome activation by WT secretant, BS from *ΔlasB* mutant, which is devoid of elastase expression, was examined. Treatment with *ΔlasB* secretant partially restored procaspase-1 in the supernatant but caused no caspase-1 processing (Figure 3F). This finding further supports that WT secretant is unable to promote inflammasome activation.

### BS from QS Mutant *P. aeruginosa* Triggers NLRC4 Inflammasome Activation and Assembly

**Figure 4:**
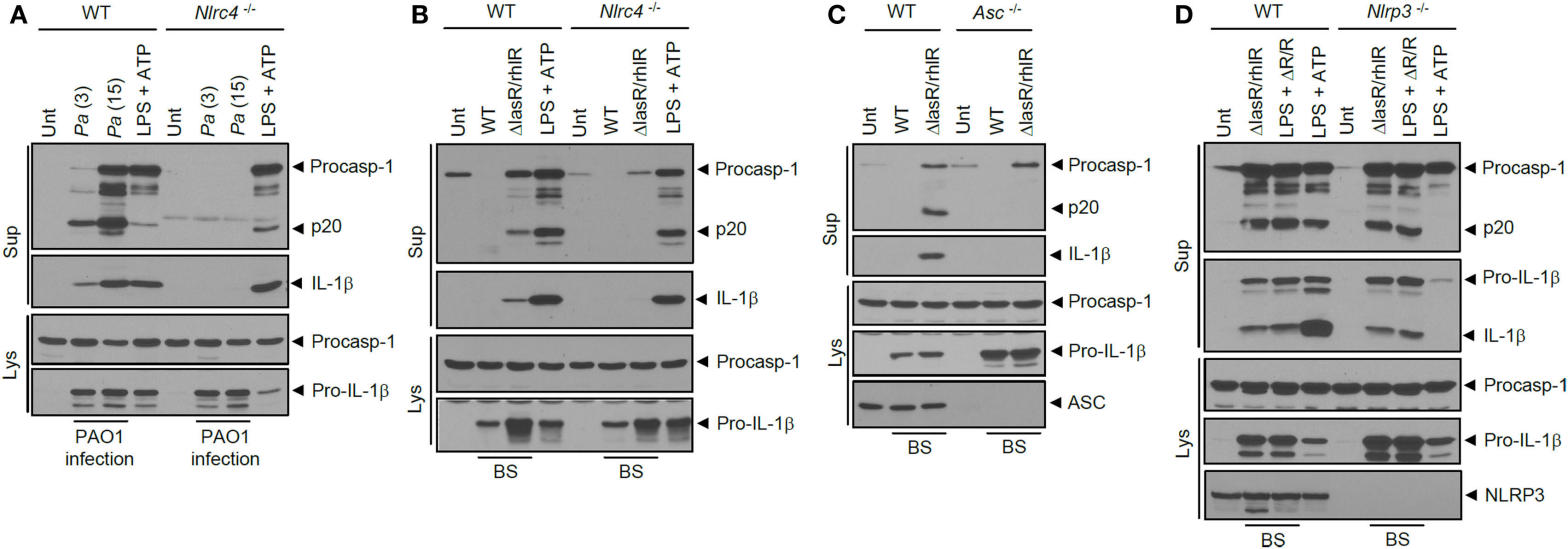
**NLRC4 inflammasome activation by quorum sensing-defective *Pseudomonas aeruginosa* (Pa) secretant**. **(A)** Wild-type (WT) or *Nlrc4^−/−^* mouse bone marrow-derived macrophages (BMDMs) were untreated or infected for 3 h with WT *Pa* at the indicated multiplicity of infection, or treated with lipopolysaccharide (LPS) (0.25 μg/ml, 3 h), followed by treatment with ATP (2.5 mM, 45 min). **(B)** WT or *Nlrc4^−/−^* mouse BMDMs were untreated (Unt) or treated with WT or *ΔlasR/rhlR P. aeruginosa* secretants for 6 h, or primed with LPS (0.25 μg/ml, 3 h), followed by treatment with ATP (2 mM, 40 min). **(C)** WT or *Asc^−/−^* mouse immortalized BMDMs were treated with WT or *ΔlasR/rhlR* secretants for 6 h. **(D)** WT or *Nlrp3^−/−^* mouse BMDMs were treated with *ΔlasR/rhlR* (ΔR/R) secretants in the presence or absence of LPS (0.25 μg/ml) for 6 h, or primed with LPS (3 h), followed by treatment with ATP (2.5 mM, 40 min). **(A–D)** Culture supernatants (Sup) or cellular lysates (Lys) were immunoblotted with the indicated antibodies. BS, bacterial secretant.

Next, we examined which sensor molecule is implicated in inflammasome activation by *ΔlasR/rhlR* secretant. In accordance with previous studies, *P. aeruginosa* infection in BMDMs resulted in NLRC4-dependent inflammasome activation (Figure 4A). Likewise, inflammasome activation by *ΔlasR/rhlR* secretant was abrogated in *Nlrc4^−/−^* BMDMs (Figure 4B), indicating that *ΔlasR/rhlR* secretant triggered NLRC4-dependent inflammasome activation. A deficiency of ASC also diminished the caspase-1 activation and IL-1β secretion by *ΔlasR/rhlR* secretant (Figure 4C). By contrast, robust inflammasome activation was observed in *Nlrp3*-deficient BMDMs in response to *ΔlasR/rhlR* secretant (Figure 4D). Therefore, our data demonstrated that *ΔlasR/rhlR* secretant promotes NLRC4-mediated, but not NLRP3-mediated inflammasome activation.

**Figure 5:**
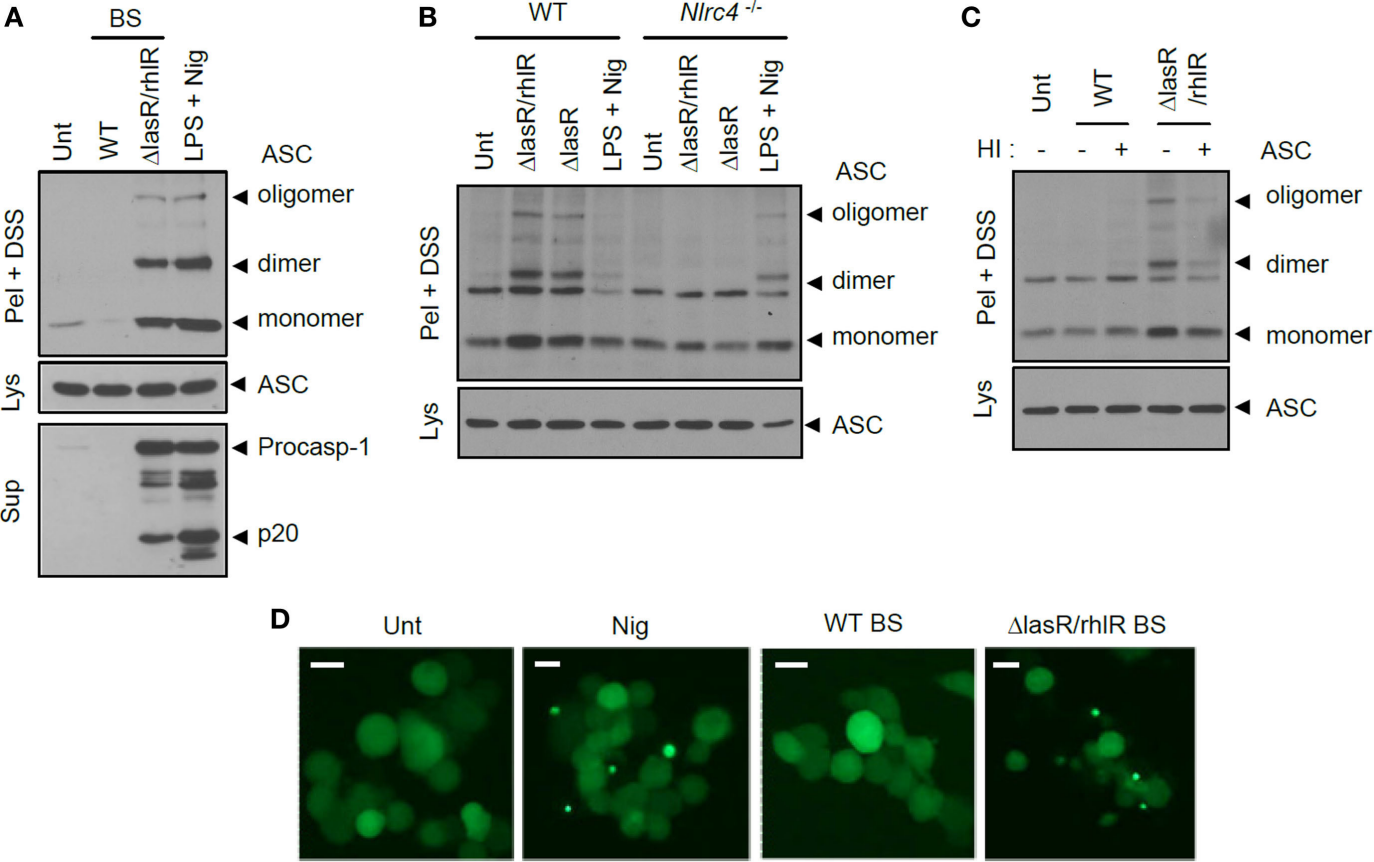
**Quorum sensing-defective *Pseudomonas aeruginosa* secretant triggers NLRC4-dependent oligomerization of ASC**. **(A)** Mouse bone marrow-derived macrophages (BMDMs) were untreated or treated with wild-type (WT) or Δ*lasR/rhlR* secretants for 6 h, or primed with lipopolysaccharide (LPS) (3 h), followed by treatment with nigericin (5 μM, 45 min). **(B)** WT or *Nlrc4^−/−^* mouse BMDMs were treated with *ΔlasR/rhlR* or *ΔlasR* secretants for 6 h, or primed with LPS, followed by treatment with nigericin. **(C)** Mouse BMDMs were treated with intact or heat-inactivated WT or *ΔlasR/rhlR* secretants. HI, heat inactivation. **(A–C)** Culture supernatants (Sup) or cellular lysates (Lys) were immunoblotted with the indicated antibodies. Disuccinimidyl suberate (DSS)-cross-linked pellets (Pel + DSS) were immunoblotted with ASC antibody. **(D)** THP-1-ASC-GFP cells were untreated or treated with WT or *ΔlasR/rhlR* secretants (8 h), or with nigericin (Nig, 5 μM, 40 min). Cells were then observed by confocal microscopy. Scale bars, 10 μm. Data shown are a representative image from eight independent samples.

Previously, it was shown that *Salmonella* infection triggered ASC oligomerization, considered as an essential event of inflammasome assembly (31, 32). Similarly, *P. aeruginosa* infection caused a robust oligomerization of ASC as determined by a DSS-mediated cross-linking assay (Figure S3 in Supplementary Material). The secretant of *ΔlasR/rhlR P. aeruginosa*, but not of WT, also triggered the formation of ASC dimeric and oligomeric structures only in the presence of NLRC4 (Figures 5A,B). The failure of WT secretant to induce ASC oligomerization further supports the hypothesis that it could not activate inflammasome signaling regardless of protease-mediated protein degradation. Moreover, even heat-inactivated WT secretant failed to promote the oligomerization of ASC inside cells (Figure 5C). Consistent with these data, BS from *ΔlasR/rhlR* mutant *P. aeruginosa*, but not from WT, triggered the formation of speck-like aggregates of ASC in human monocytic THP-1 cells expressing ASC-GFP (Figure 5D). These findings collectively demonstrate that the *ΔlasR/rhlR* mutant secretant, but not WT secretant, promotes the assembly and activation of NLRC4 inflammasome.

### Extracellular Flagellin Mediates Inflammasome Activation by QS Mutant Secretant

**Figure 6:**
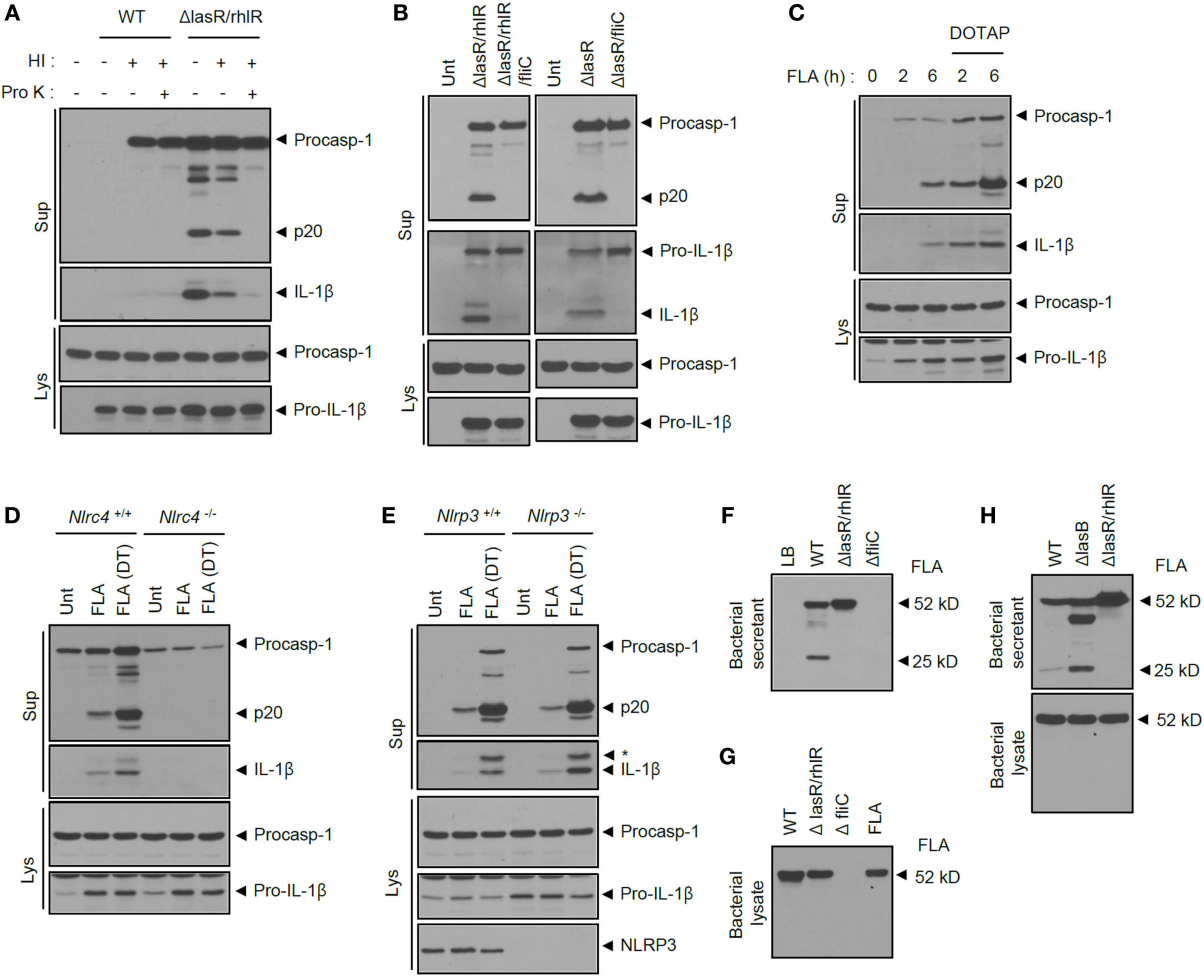
**Extracellular flagellin is critical for inflammasome activation by quorum sensing-defective *Pseudomonas aeruginosa* secretant**. **(A)** Mouse bone marrow-derived macrophages (BMDMs) were untreated or treated with wild-type (WT) or *ΔlasR/rhlR P. aeruginosa* secretants with or without heat inactivation (HI) and proteinase K (Pro K) treatment as indicated. **(B)** Mouse BMDMs were treated for 6 h with *ΔlasR/rhlR, ΔlasR/rhlR/fliC, ΔlasR*, or *ΔlasR/fliC* secretants as indicated. **(C)** Mouse BMDMs were treated with *P. aeruginosa* flagellin (FLA, 0.25 μg) for 2 or 6 h with or without pre-mixing with DOTAP liposomal transfection reagent. **(D,E)** WT, *Nlrc4^−/−^* **(D)** or *Nlrp3^−/−^* **(E)** mouse BMDMs were treated with flagellin (0.25 μg) with or without pre-mixing with DOTAP (DT) reagent for 6 h. **(A–E)** Culture supernatants (Sup) or cellular lysates (Lys) were immunoblotted with the indicated antibodies. **(F)** Bacterial secretants (BSs) from WT, Δ*lasR/rhlR* or Δ*fliC P. aeruginosa* cultures were immunoblotted with anti-flagellin antibody. **(G)** WT, *ΔlasR/rhlR*, and *ΔfliC P. aeruginosa* bacterial lysates or purified *P. aeruginosa* flagellin (FLA) were immunoblotted with anti-flagellin antibody. **(H)** BSs or bacterial lysates from WT, Δ*lasB*, or Δ*lasR/rhlR P. aeruginosa* cultures were immunoblotted with anti-flagellin antibody.

To further provide molecular insight into the inflammasome-activating capability of *ΔlasR/rhlR* secretant, we first examined whether specific protein components of BS drive inflammasome activation. Heat inactivation of *ΔlasR/rhlR* secretant significantly reduced caspase-1 activation and IL-1β secretion (Figure 6A; Figure S4A in Supplementary Material). This is consistent with the above results in Figures 3C,D, and these findings demonstrate that heat treatment significantly impairs BS-promoted inflammasome activation. Moreover, proteinase K treatment of *ΔlasR/rhlR* secretant completely abolished the inflammasome activation (Figure 6A; Figure S4A in Supplementary Material). These data strongly indicate that unidentified proteins in the secretant could be responsible for the secretant-triggered inflammasome activation. According to previous report, cytosolic presence of bacterial flagellin is critical to induce NLRC4 inflammasome activation upon *P. aeruginosa* infection (21). Related to this finding, we found that BS from the *ΔfliC* mutant lacking FliC flagellin was unable to promote inflammasome activation (Figure S4B in Supplementary Material). Intriguingly, the ablation of FliC in *ΔlasR/rhlR* or *ΔlasR P. aeruginosa* completely abolished the inflammasome activation by *ΔlasR/rhlR* or *ΔlasR* BS (Figure 6B), suggesting that flagellin in *ΔlasR/rhlR* secretant might be the main stimulus for inflammasome activation.

To verify this possibility that extracellular flagellin released from *P. aeruginosa* could trigger inflammasome activation, we treated BMDMs with purified flagellin from *P. aeruginosa*. Previous reports showed that intracellular delivery of flagellin using a liposomal agent such as DOTAP is essential for activation of the NLRC4 inflammasome (3, 33). Treatment of flagellin for 1–3 h failed to activate caspase-1 and IL-1β secretion in BMDMs (3, 21). However, in our study, 6-h incubation of flagellin in the extracellular medium was able to promote caspase-1 activation and IL-1β secretion (Figure 6C). This extracellular flagellin-triggered inflammasome activation depended on NLRC4 but not NLRP3 (Figures 6D,E). To explain WT secretant’s inability to activate the inflammasome, the presence of flagellin was assayed in the secretant from *P. aeruginosa* cultures. Of interest, intact flagellin was observed in the BS of the *ΔlasR/rhlR* mutant, whereas flagellin was severely impaired in the WT secretant (Figure 6F). Indeed, flagellin levels were markedly reduced in WT secretant, but not in QS-defective mutants secretant (Figure S4C in Supplementary Material). By contrast, no degradation of flagellin was observed in the bacterial lysate of WT *P. aeruginosa* (Figure 6G), indicating that secreted flagellin could be degraded by bacterial proteases in the secretant rather than within the cells. The degradation of flagellin was also observed, but slightly reduced in *ΔlasB* mutant, suggesting that both elastase and other protease are responsible for the impairment of flagellin (Figure 6H). These data demonstrate that QS-dependent production of protease from WT *P. aeruginosa* culture led to degradation of flagellin and thereby eliminated its ability to activate the NLRC4 inflammasome.

### QS-Regulated Virulence Factor Pyocyanin Attenuates Inflammasome Activation

**Figure 7:**
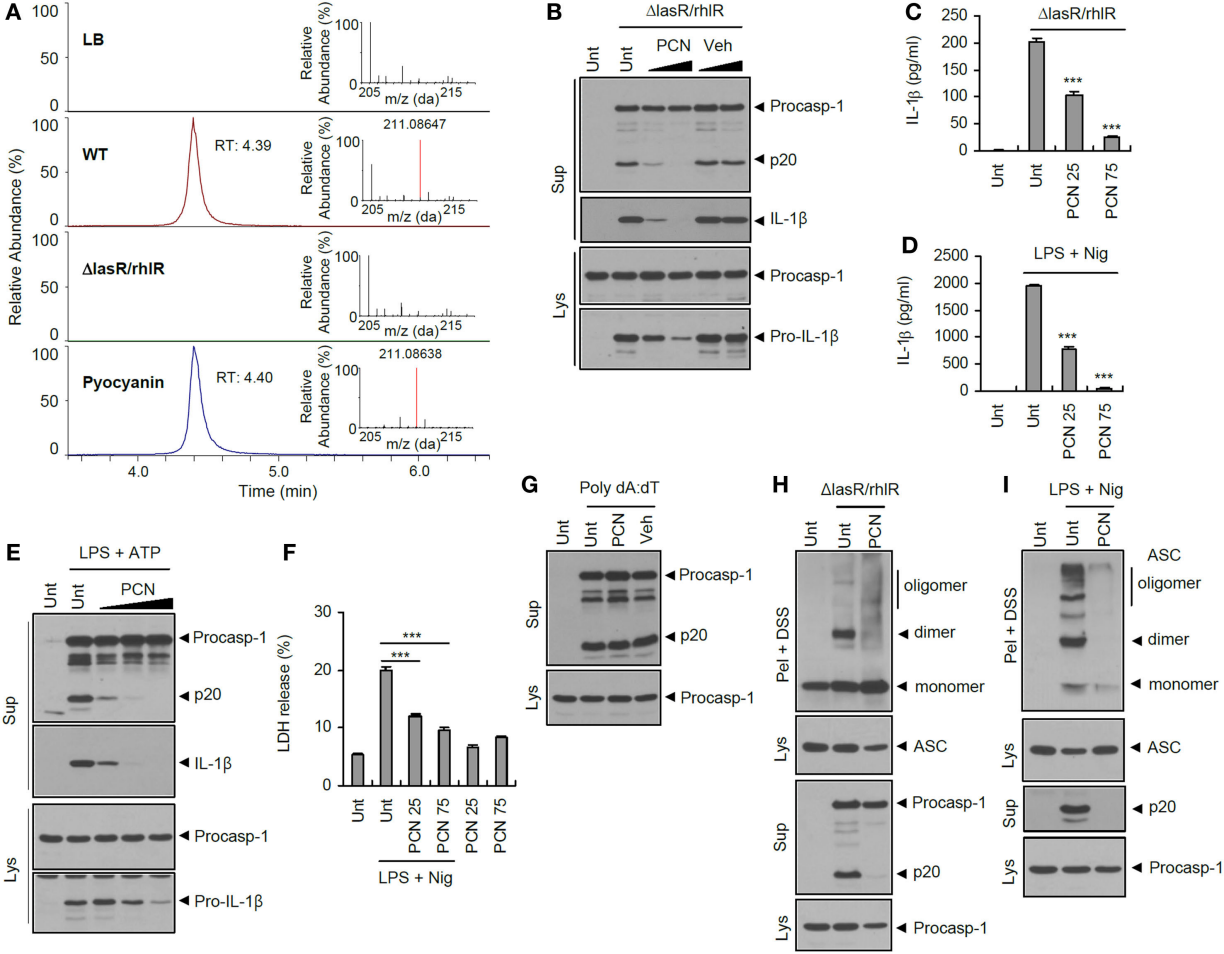
**Suppression of inflammasome activation by quorum sensing-regulated product pyocyanin**. **(A)** Extracted ion chromatogram for *m/z* 211.08647 (tolerance; 5 ppm) and mass spectra (RT 4.3–4.5) of each samples and standard pyocyanin in full scan mode. **(B)** Mouse bone marrow-derived macrophages (BMDMs) were treated with *ΔlasR/rhlR Pseudomonas aeruginosa* secretants in the presence of pyocyanin (PCN, 60 or 120 μM) or DMSO vehicle for 6 h. **(C)** Mouse BMDMs were treated with *ΔlasR/rhlR* secretant in the presence of pyocyanin (25 or 75 μM, 30 min before secretant) for 6 h. **(D)** Mouse BMDMs were primed with lipopolysaccharide (LPS) (0.25 μg/ml, 3 h) in the presence of pyocyanin (25 or 75 μM, 30 min before LPS), followed by treatment with nigericin (Nig, 5 μM, 45 min). **(C,D)** The levels of interleukin-1 beta (IL-1β) were quantified by ELISA in BMDM supernatants. Asterisks indicate significant differences compared with *ΔlasR/rhlR*-treated (C) or LPS/nig-treated (D) group (*n* = 3). **(E)** Mouse BMDMs were primed with LPS (0.25 μg/ml, 3 h) in the presence of pyocyanin (20, 50, and 100 μM) pretreatment (30 min before LPS), followed by treatment with ATP (2 mM, 45 min). **(F)** Mouse BMDMs were primed with LPS (0.25 μg/ml, 3 h) in the presence of pyocyanin pretreatment (25 or 75 μM, 30 min before LPS), followed by treatment with nigericin (5 μM, 30 min). Extracellular release of lactate dehydrogenase (LDH) was assayed. Asterisks indicate significant differences (*n* = 3). **(G)** Mouse BMDMs were untreated or transfected with poly dA:dT (1.5 μg, 5 h) in the presence of pyocyanin (50 μM, 30 min pretreatment). **(H)** Mouse BMDMs were treated with *ΔlasR/rhlR* secretants in the presence of pyocyanin (120 μM) for 6 h. **(I)** Mouse BMDMs were primed with LPS (0.25 μg/ml, 3 h) in the presence of pyocyanin (120 μM, 30 min before LPS), followed by treatment with nigericin (5 μM, 40 min). **(B,E,G–I)** Culture supernatants (Sup) or cellular lysates (Lys) were immunoblotted with the indicated antibodies. Disuccinimidyl suberate (DSS)-cross-linked pellets (Pel + DSS) were immunoblotted with ASC antibody. Statistical significance was determined by one-way analysis of variance with a Bonferroni post-test (***P < 0.001).

To further delineate the potential contribution of QS to inflammasome activation, metabolomic analysis was performed with BSs from WT and *ΔlasR/rhlR P. aeruginosa* using liquid chromatography–mass spectrometry. Unfortunately, under our experimental conditions, we did not identify any candidate molecule that was only present in *ΔlasR/rhlR* secretant, but not in WT secretant. However, pyocyanin was identified as the sole candidate molecule present in WT secretant but not in *ΔlasR/rhlR* secretant (Figure 7A; Figures S5A,B in Supplementary Material). Pyocyanin is one of the well-known QS-dependent major virulence factors of *P. aeruginosa* (17, 18) and was reported to damage host cells *via* its oxidative stress-based cytotoxicity (34, 35). Thus, we tested a potential role of pyocyanin in the inflammasome activation. Of note, pyocyanin remarkably suppressed the inflammasome activation and IL-1β secretion triggered by *ΔlasR/rhlR* secretant (Figures 7B,C). Furthermore, pyocyanin treatment resulted in a strong suppression of NLRP3-mediated caspase-1 activation and IL-1β secretion upon LPS/ATP or LPS/nigericin stimulation (Figures 7D,E). Pyocyanin also significantly attenuated LPS/nigericin-triggered cell death of BMDMs (Figure 7F). This might be due to reduced caspase-1 activation by pyocyanin. However, pyocyanin failed to affect poly dA:dT-triggered AIM2 inflammasome activation (Figure 7G). Supporting these observations, pyocyanin clearly reduced NLRC4- or NLRP3-dependent ASC oligomerization in response to *ΔlasR/rhlR* secretant or LPS/nigericin treatment (Figures 7H,I). These findings demonstrate that the QS-dependent virulence factor pyocyanin could suppress the activation of both the bacterial infection-promoted NLRC4 inflammasome and the host endogenous danger signals-triggered NLRP3 inflammasome.

### QS Autoinducer 3-oxo-C12-HSL Attenuates Inflammasome Activation

**Figure 8:**
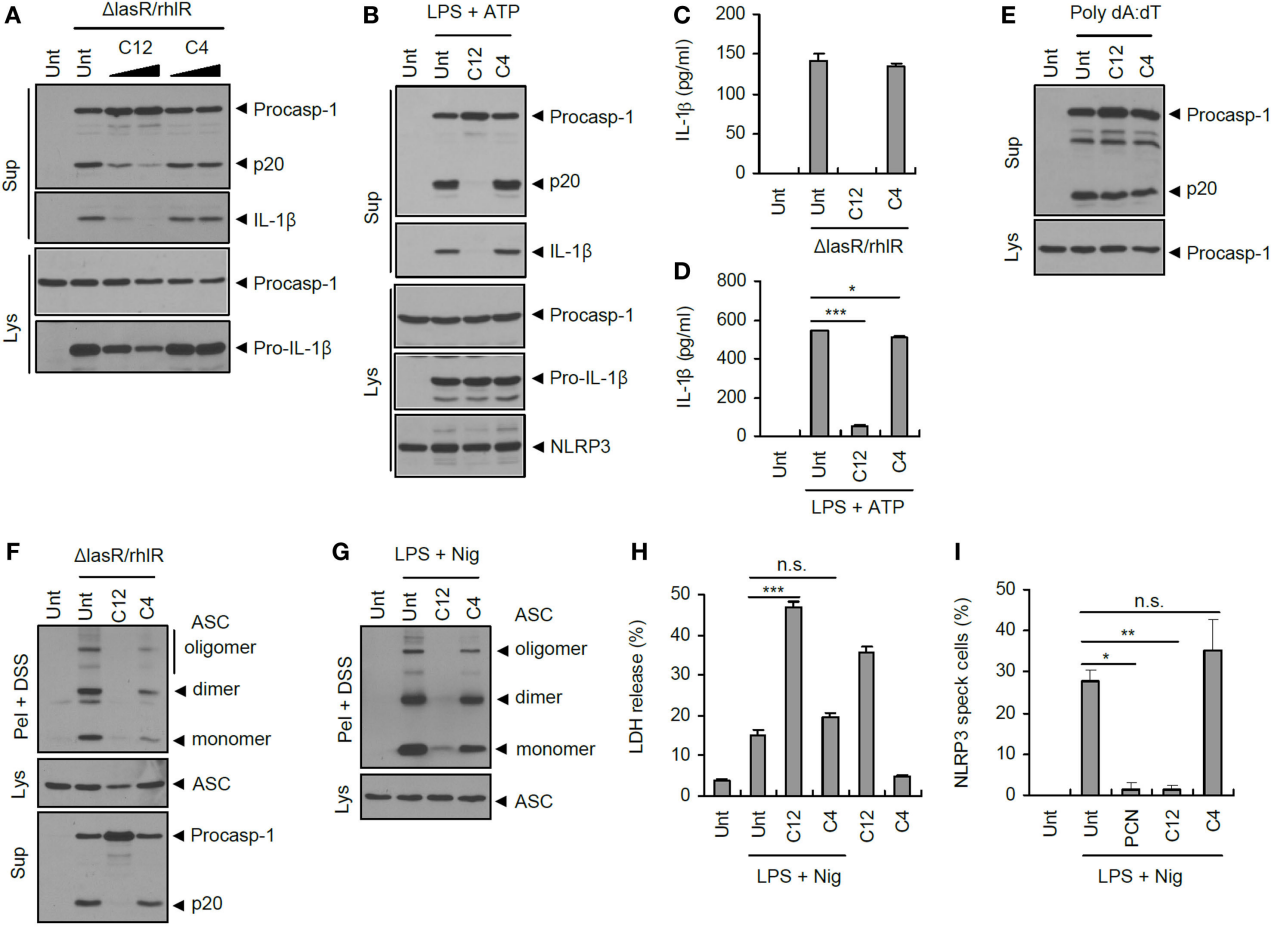
**Suppression of inflammasome activation by quorum sensing autoinducer 3-oxo-C12-HSL**. **(A)** Mouse bone marrow-derived macrophages (BMDMs) were treated with *ΔlasR/rhlR* secretants in the presence of 3-oxo-C12-HSL (C12) or C4-HSL (C4) (30, 80 μM, 30 min pretreatment) for 6 h. **(B)** Mouse BMDMs were primed with lipopolysaccharide (LPS) (0.25 μg/ml, 3 h) in the presence of C12-HSL or C4-HSL pretreatment (40 μM), followed by treatment with ATP (2 mM, 30 min). **(C)** Mouse BMDMs were treated with *ΔlasR/rhlR* secretants in the presence of 3-oxo-C12-HSL or C4-HSL pretreatment (80 μM) for 6 h. **(D)** Mouse BMDMs were primed with LPS (0.25 μg/ml, 3 h) in the presence of 3-oxo-C12-HSL or C4-HSL (40 μM, 30 min before LPS) for 3 h, followed by treatment with ATP (2 mM, 40 min). **(C,D)** The levels of interleukin-1 beta (IL-1β) were quantified by ELISA in BMDM supernatants. Asterisks indicate significant difference (*n* = 3). **(E)** Mouse BMDMs were untreated or transfected with poly dA:dT (1.5 μg, 5 h) in the presence of C12-HSL or C4-HSL (50 μM, 30 min pretreatment). **(F)** Mouse BMDMs were treated with *ΔlasR/rhlR* secretants in the presence of 3-oxo-C12-HSL or C4-HSL (80 μM, 30 min pretreatment) for 6 h. **(G)** Mouse BMDMs were primed with LPS (0.25 μg/ml, 3 h) in the presence of 3-oxo-C12-HSL or C4-HSL (40 μM, 30 min before LPS), followed by treatment with nigericin (5 μM, 30 min). **(H)** Mouse BMDMs were primed with LPS (0.25 μg/ml, 3 h) in the presence of 3-oxo-C12-HSL or C4-HSL (20 μM, 30 min before LPS), followed by treatment with nigericin (5 μM, 30 min). Extracellular release of lactate dehydrogenase (LDH) was assayed. Asterisk indicates significant differences (*n* = 3). **(I)** NLRP3-GFP-expressing BMDMs were primed with LPS (0.25 μg/ml, 3 h) in the presence of pyocyanin (PCN), 3-oxo-C12-HSL, or C4-HSL (50 μM, 30 min before LPS), followed by treatment with nigericin (5 μM, 40 min). Cells were observed by confocal microscope, and NLRP3 speck-containing cells were counted. Asterisks indicate significant difference (*n* = 3). **(A,B,E–G)** Culture supernatants (Sup) or cellular lysates (Lys) were immunoblotted with the indicated antibodies. Disuccinimidyl suberate (DSS)-cross-linked pellets (Pel + DSS) were immunoblotted with ASC antibody. Statistical significance was determined by one-way analysis of variance with a Bonferroni post-test (*P < 0.05; ***P < 0.001).

Bacterial QS systems use acylated homoserine lactones as a QS signaling regulator, called an autoinducer. In the case of *P. aeruginosa*, two signaling molecules—3-oxo-C12-HSL, produced by LasI, and C4-HSL, produced by RhlI—are employed for bacterial communication to control the expression of many virulence factors (18). So far, these QS autoinducers have never been examined for their potential effect on inflammasome signaling. Interestingly, 3-oxo-C12-HSL, but not C4-HSL, significantly dampened inflammasome activation triggered by *ΔlasR/rhlR* secretant (Figure 8A). Moreover, 3-oxo-C12-HSL completely blocked the NLRP3 inflammasome activation in response to LPS/ATP stimulation (Figure 8B). This *P. aeruginosa* autoinducer 3-oxo-C12-HSL also strongly attenuated IL-1β secretion upon *ΔlasR/rhlR* secretant or LPS/ATP treatment (Figures 8C,D). Similar to pyocyanin, two *P. aeruginosa* autoinducers showed no effect on the AIM2-dependent caspase-1 activation triggered by poly dA:dT transfection (Figure 8E), indicating that 3-oxo-C12-HSL could suppress NLRC4 and NLRP3 inflammasome signaling. Supporting these findings, 3-oxo-C12-HSL diminished the NLRC4- and NLRP3-mediated ASC oligomerization by cross-linking experiments (Figures 8F,G). However, NLRP3 inflammasome-dependent cell death was not abolished, but rather increased by 3-oxo-C12-HSL (Figure 8H). Indeed, 3-oxo-C12-HSL showed a basal cytotoxicity to BMDMs regardless of inflammasome activity. Of notice, QS-associated factors, pyocyanin and 3-oxo-C12-HSL, significantly inhibited the oligomerization of NLRP3 in response to classical NLRP3 stimulators (Figure 8I; Figure S6 in Supplementary Material). These data suggest that the suppression of inflammasome signaling by two QS-related factors might be the upstream event of oligomerization of inflammasome sensor molecule such as NLRP3. These findings collectively demonstrate that two QS-related products, pyocyanin and 3-oxo-C12-HSL, are *bona fide* inhibitors of inflammasome activation, and that bacterial QS could provide an evasion mechanism to avoid the host inflammasome defense system.

## Discussion

Despite the recent advances in knowledge regarding bacterial recognition pathways leading to inflammasome activation, bacterial evasion of inflammasome activation remains still poorly understood. Moreover, most studies focus on the host cell’s response after uptake of bacteria. Thus, the potent role of extracellular bacteria-secreted factors during pathogenesis has not been fully investigated. In the present study, we attempted to dissect the role of *P. aeruginosa* factors that are secreted from that of internalized bacteria using bacteria-free secretant. Here, we present novel evidence demonstrating that *P. aeruginosa* makes use of bacterial QS-dependent secretant as an inflammasome evasion strategy.

**Figure 9:**
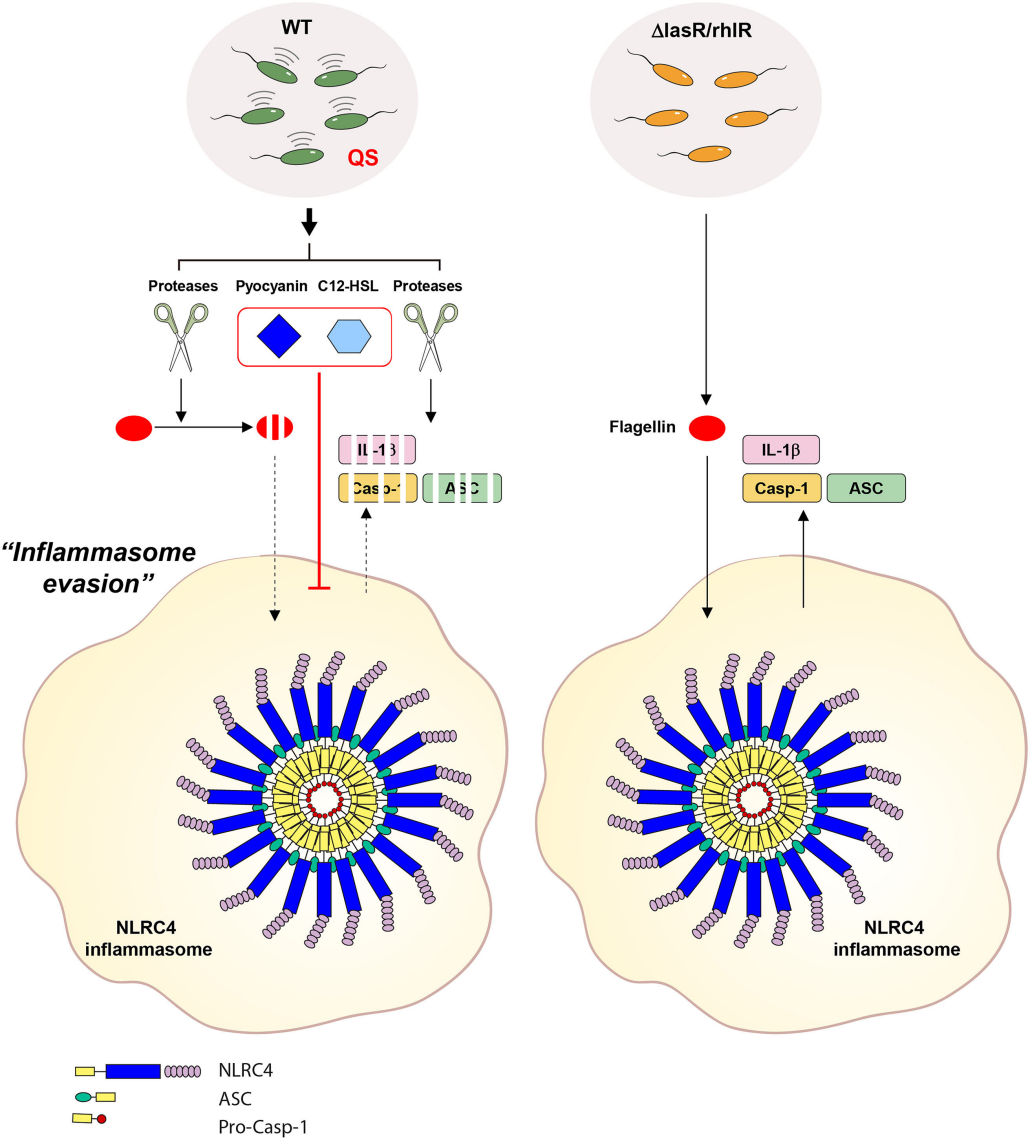
**Proposed mechanism for the contribution of *Pseudomonas aeruginosa* secretant to NLRC4 inflammasome activation**. Wild-type (WT) *P. aeruginosa* secrets quorum sensing (QS)-regulated factors such as proteases, pyocyanin and 3-oxo-C12-HSL in a QS-dependent manner. In the extracellular space, secreted proteases degrade inflammasome-associated molecules from host cells. Proteases also degrade extracellular flagellin to impair its potent NLRC4-stimulating ability. Of note, extracellular flagellin originating from *P. aeruginosa* can promote the activation of the NLRC4 inflammasome without bacterial invasion into host cells. Furthermore, two QS factors, pyocyanin and C12-HSL, suppress the activation of the NLRC4 inflammasome. QS-defective *P. aeruginosa* fail to produce the QS-regulated factors, mentioned above. Therefore, QS mutants could lead to deregulated inflammasome activation in certain contexts.

Upon reaching a threshold density of bacterial growth, the bacterial QS system initiates the expression of an array of virulence determinants using AHLs as an autoinducer (36). Consequently, this regulated expression of virulence genes is thought to confer a selective advantage to bacteria over host defenses and to impair host protective functions in various ways (17, 36). For example, *P. aeruginosa*-secreted proteases, whose expression was greatly increased by the QS system, degrade many host defense proteins such as complement, surfactant, and ECM proteins (11). In addition, *P. aeruginosa* elastase is known to lead to degradation of pro-inflammatory cytokines such as IL-6 or IL-8 to avoid host immune defense (37). A recent report also showed that thrombin cleaved by *P. aeruginosa* elastase attenuated TLR-mediated pro-inflammatory responses (38). In correlation with these studies, our results clearly demonstrate that *P. aeruginosa* proteases degrade inflammasome components including caspase-1 and ASC, as well as pro-inflammatory cytokines in the extracellular space (Figure 9). Thus, QS-mediated production of proteases by *P. aeruginosa* provides the bacterial community an opportunity to debilitate the host’s innate immune protection.

The *P. aeruginosa* QS system also controls the production of redox-active phenazine derivatives such as pyocyanin (17). We detected pyocyanin in WT, but not QS-defective mutant, *P. aeruginosa* secretant using a metabolomic analysis. Pyocyanin, one of the major toxins secreted by *P. aeruginosa*, was shown to damage or kill host target cells mainly by inducing oxidative stress (34, 39). Pyocyanin also plays an important role in the pathogenesis of cystic fibrosis upon *P. aeruginosa* infection (40). Of particular interest, the level of pyocyanin is elevated up to 100 μM in *P. aeruginosa*-infected patients with cystic fibrosis (41). Moreover, virulence was attenuated in pyocyanin-deficient *P. aeruginosa*-infected mice (42), indicating that pyocyanin is one of the major virulence factors of *P. aeruginosa*. In terms of its contribution to host inflammatory response, pyocyanin can exert a pro-inflammatory role based on its reactive oxygen species-producing ability (39). By contrast, pyocyanin-deficient *P. aeruginosa* infection caused increased inflammation in the bronchoalveolar lavage of mice (35), suggesting potent anti-inflammatory function of pyocyanin. In line with these data, our results present the first evidence that pyocyanin could suppress NLRC4- and even NLRP3-mediated inflammasome activation. Although the underlying mechanism by which pyocyanin inhibits the activation of inflammasome signaling remains to be elucidated, our data suggest the novel concept that *P. aeruginosa* could attenuate inflammasome activation through QS-dependent production of pyocyanin.

In addition to the contribution of proteases and pyocyanin to host inflammasome signaling, we also unveiled a novel function of *P. aeruginosa* QS autoinducer C12-HSL in suppression of inflammasome activation. An anti-inflammatory role of C12-HSL was previously reported. C12-HSL inhibits LPS-triggered production of pro-inflammatory cytokines, such as TNF-α and MCP-10, in peritoneal macrophages and BMDMs (43–45). Furthermore, C12-HSL, but not C4-HSL, causes apoptosis in BMDMs, which impairs the host immune defense against *P. aeruginosa* infection (46). These observations indicate that *P. aeruginosa* C12-HSL can not only trigger bacterial virulence gene expression as a QS autoinducer but also attenuate host innate immune defense. The present data provide additional insight into the anti-inflammatory role of C12-HSL in diminishing the activation of NLRP3 and NLRC4 inflammasome pathways. These findings suggest that the *P. aeruginosa* QS system could dampen host inflammasome-mediated protection *via* QS-associated products such as pyocyanin and C12-HSL (Figure 9).

The primary role of NLRC4 inflammasome signaling in response to *P. aeruginosa* infection is to control bacterial replication. Previous studies showed that inflammasome-deficient mice lacking NLRC4, caspase-1, IL-1β, or IL-1 receptor resulted in elevated bacterial burden and tissue damages upon *P. aeruginosa* infection (20, 47–49). Pattern-recognition host cells detect key virulence determinants of *P. aeruginosa*, such as flagellin or T3SS protein PscC, leading to NLRC4 inflammasome activation (21, 50, 51). In terms of bacterial evasion of host innate defense, *P. aeruginosa* major exotoxin ExoU was shown to attenuate NLRC4 inflammasome activation, likely through its phospholipase activity (47). Also, alkaline protease AprA, a QS-dependent secreted protease, degrades bacterial monomeric flagellin to evade TLR5-mediated immune responses (52). In close association with these previous findings, our data suggest that QS-dependent production of *P. aeruginosa* proteases degrade extracellular flagellin, leading to the impairment of flagellin-triggered inflammasome activation. Until now, it was generally accepted that cytosolic delivery of flagellin through bacterial secretion system or *in vitro* liposomal transfection reagent is prerequisite for inflammasome activation (3, 33). Intriguingly, our study shows that extracellular secreted flagellin alone can induce inflammasome activation. One possible explanation for this discrepancy could be the different treatment times. Previous studies examined the inflammasome activation in BMDMs only upon less than 3 h *Salmonella* flagellin treatment (3, 21); however, we obtained inflammasome activation after 6 h treatment of *P. aeruginosa* flagellin. Nevertheless, we still believe that the delivery of flagellin into cytosol is required to activate inflammasome. Further study will be needed to clarify how extracellular flagellin is able to penetrate host cell membrane.

In contrast to its protective role, inflammasome activation has also been proposed to exacerbate host tissue damages in response to *P. aeruginosa* infection (53, 54). It is possible that the initial activation of inflammasome signaling is preferable for host defense, especially upon acute *P. aeruginosa* infection. However, it is highly plausible that persistently dysregulated inflammasome activation triggered by *P. aeruginosa* may cause host tissue damage. Bacterial QS is thought to significantly enhance pathogenicity by promoting the acute expression of diverse virulence factors. Paradoxically, QS-defective strains such as LasR-lacking *P. aeruginosa* were highly represented in the clinical isolates from cystic fibrosis patients (55) and these patients are associated with exacerbated lung pathology (56). Although it is not completely understood why QS mutant *P. aeruginosa*, considered a strain with reduced virulence, were present in cystic fibrosis patients, our data provide a possible explanation. Specifically, QS-defective mutants could promote hyper-activation of inflammasome signaling by disarming inflammasome-suppressing machinery, which might lead to the exacerbated pathology of cystic fibrosis patients. Of note, QS products pyocyanin and C12-HSL effectively suppressed NLRP3 inflammasome activation triggered by classical NLRP3 stimulators. Given that *P. aeruginosa* flagellin was also proposed to induce NLRP3 inflammasome activation (57), chronic infection with *P. aeruginosa* could lead to excessive NLRP3 inflammasome activation. In this regard, QS-defective mutants are implicated in the dysregulation of NLRP3 inflammasome activation that accounts for the severe pathology of cystic fibrosis. Further studies will be needed to reveal the molecular mechanisms linking *P. aeruginosa* QS to NLRP3 inflammasome signaling. On the basis of our data, *P. aeruginosa* QS can be used to evade host inflammasome activation in the early stage of infection, but the absence of QS can cause excessive inflammation in the context of chronic infections. Our study also highlights the importance of extracellular bacterial secreted factors in modulating host innate immune responses.

## Author Contributions

JY designed and performed most of experiments. K-ML, SP, YC, EL, and JS performed experiments. J-HP, OS, and SY provided materials and scientific advices. J-WY supervised the entire project and wrote the manuscript.

## Conflict of Interest Statement

The authors declare that the research was conducted in the absence of any commercial or financial relationships that could be construed as a potential conflict of interest.

## Footnotes

^1^https://www.mzcloud.org.

^2^http://msbi.ipb-halle.de/MetFrag.

^3^https://pubchem.ncbi.nlm.nih.gov.
