# Supplementary material for: Bacterial Secretant from Pseudomonas aeruginosa Dampens Inflammasome Activation in a Quorum Sensing-Dependent Manner
Source: Front Immunol. 2017 Mar 27;8:333. doi: 10.3389/fimmu.2017.00333 (PMC5366846; doi:10.3389/fimmu.2017.00333)
Supplement: Supplementary file 1 [file presentation_1.pdf]

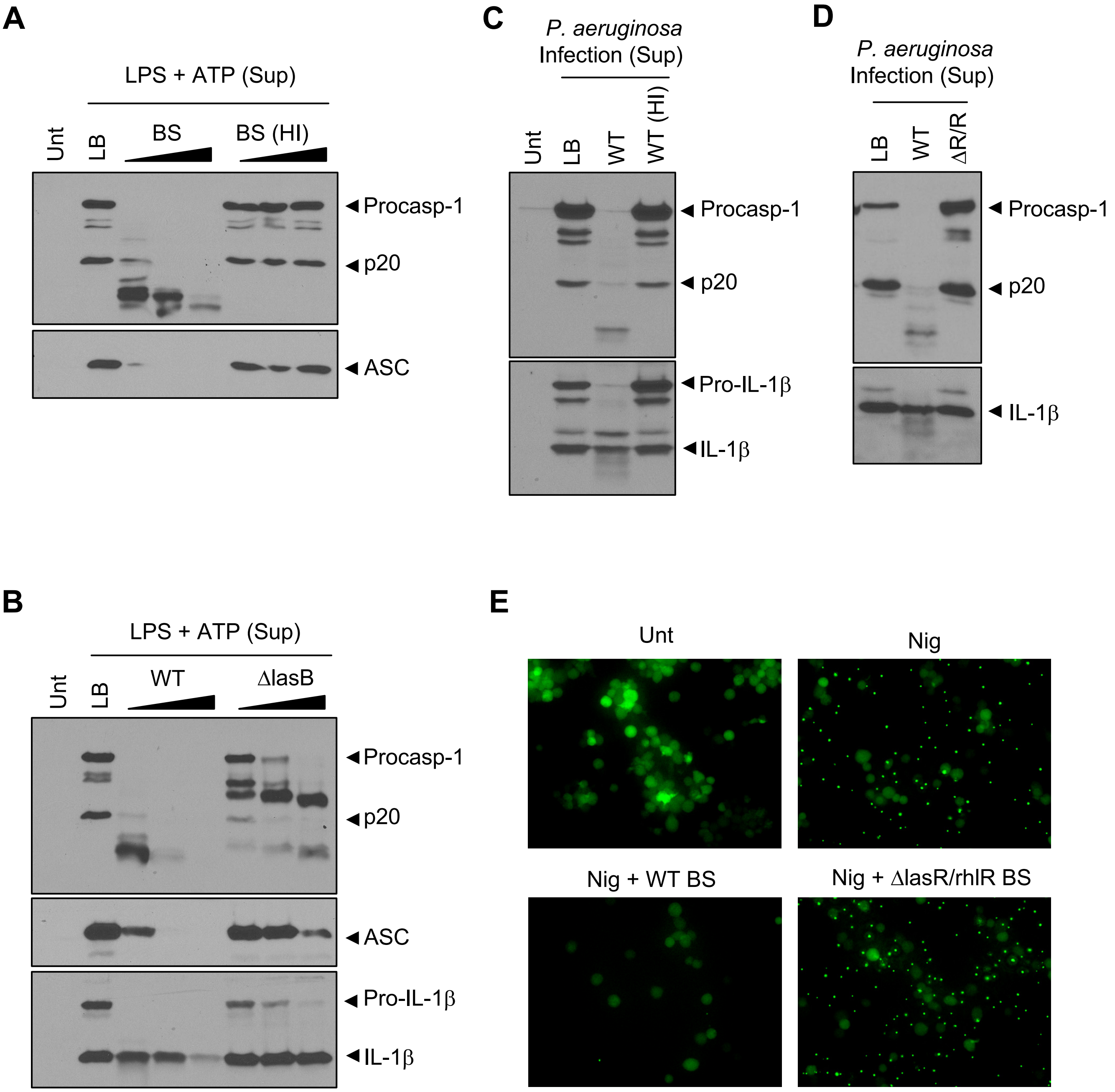

Supplementary Figure 1. Degradation of inflammasome-associated proteins by WT *P. aeruginosa* secretant. (**A,B**) LPS/ATP-stimulated BMDM culture supernatants were incubated with intact or heat-inactivated (HI) bacterial secretant (A) or WT or *ΔlasB* secretant (B) for 10, 40, or 120 min, and immunoblotted with the indicated antibodies. (**C,D**) Untreated or WT *P. aeruginosa* (MOI 3, 3 h)-infected BMDM supernatants were incubated with intact or heat-inactivated (HI) WT *P. aeruginosa* secretant for 60 min (C), or with WT or *ΔlasR/rhlR* (ΔR/R*) P. aeruginosa* secretant (D) for 60 min. (**A-D**) The mixtures were then immunoblotted with the indicated antibodies. (**E**) THP-1-ASC-GFP cells were treated with nigericin (Nig, 5 μM, 3 h) in the presence of WT or Δ*lasR/rhlR P. aeruginosa* secretant (BS). Cells were then observed by confocal microscope.

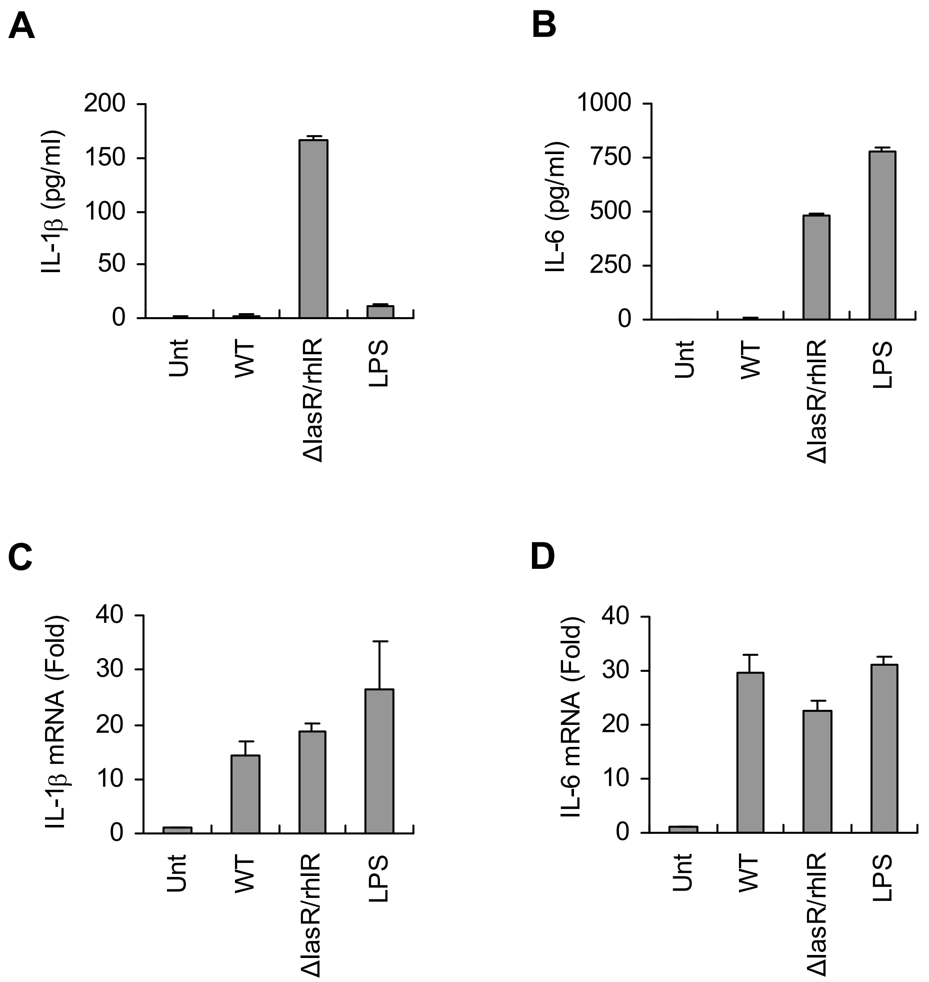

Supplementary Figure 2. Impairment of proinflammatory cytokines in the supernatants of BMDMs upon treated with wild type *P. aeruginosa* secretant. (A-D) Mouse BMDMs were treated with bacterial secretants from WT or ΔlasR/rhlR cultures for 6 h, or treated with LPS (0.25 μg/ml, 3 h). Culture supernatants were employed for the quantification of IL-1β (A) or IL-6 (B) by ELISA. Cellular levels of IL-1β (C) or IL-6 (D) mRNA were quantified by quantitative real-time PCR. (*n* = 3, A,C,D; *n* = 6, B)

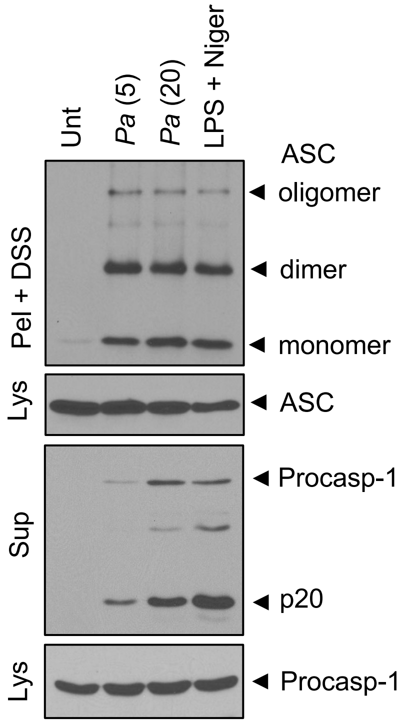

Supplementary Figure 3. ASC oligomerization by *P. aeruginosa* infection. Mouse BMDMs were infected with WT PAO1 at the indicated MOI for 3 h, or treated with LPS, followed by the treatment of nigericin (5 μM, 45 min). Culture supernatants (Sup) or cellular lysates (Lys) were immunoblotted with the indicated antibodies. DSS-crosslinked pellets (Pel + DSS) were immunoblotted with ASC antibody.

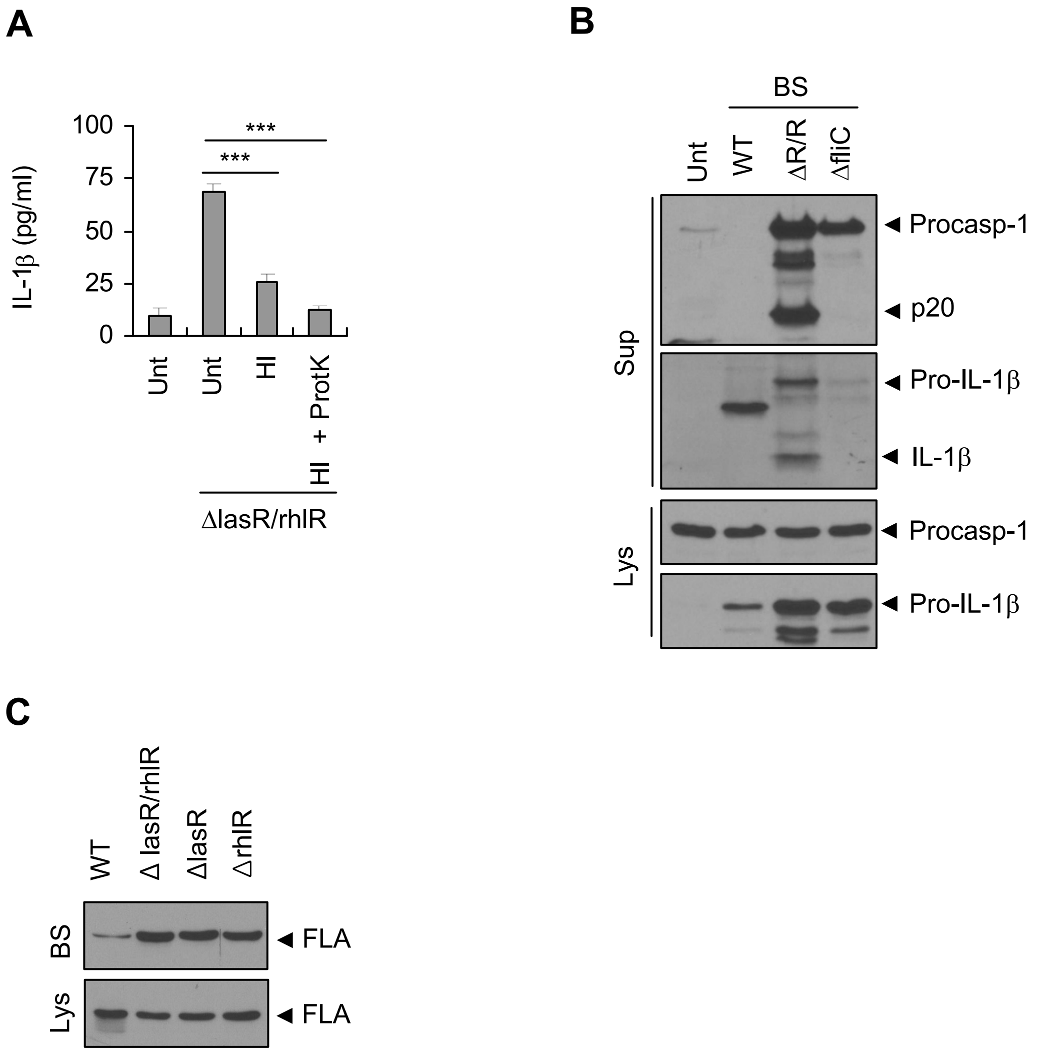

Supplementary Figure 4. Extracellular flagellin is important for the inflammasome activation in response to *ΔlasR/rhlR* secretant. (**A)** Mouse BMDMs were treated with intact, heat-inactivated (HI) or protease K-treated *ΔlasR/rhlR* secretants for 6 h. IL-1β secretion from BMDMs was quantified by ELISA. Asterisks indicate significant differences (n = 3, ***P<0.001). (**B**) Mouse BMDMs were treated with WT, *ΔlasR/rhlR* or *ΔfliC P. aeruginosa* secretants (BS) for 6 h. Culture supernatants (Sup) or cellular lysates (Lys) were immunoblotted with the indicated antibodies. (**C**) WT, *ΔlasR/rhlR, ΔlasR* or *ΔrhlR P. aeruginosa* secretants (BS) or bacterial lysates (Lys) were immunoblotted with anti-flagellin antibody.

A

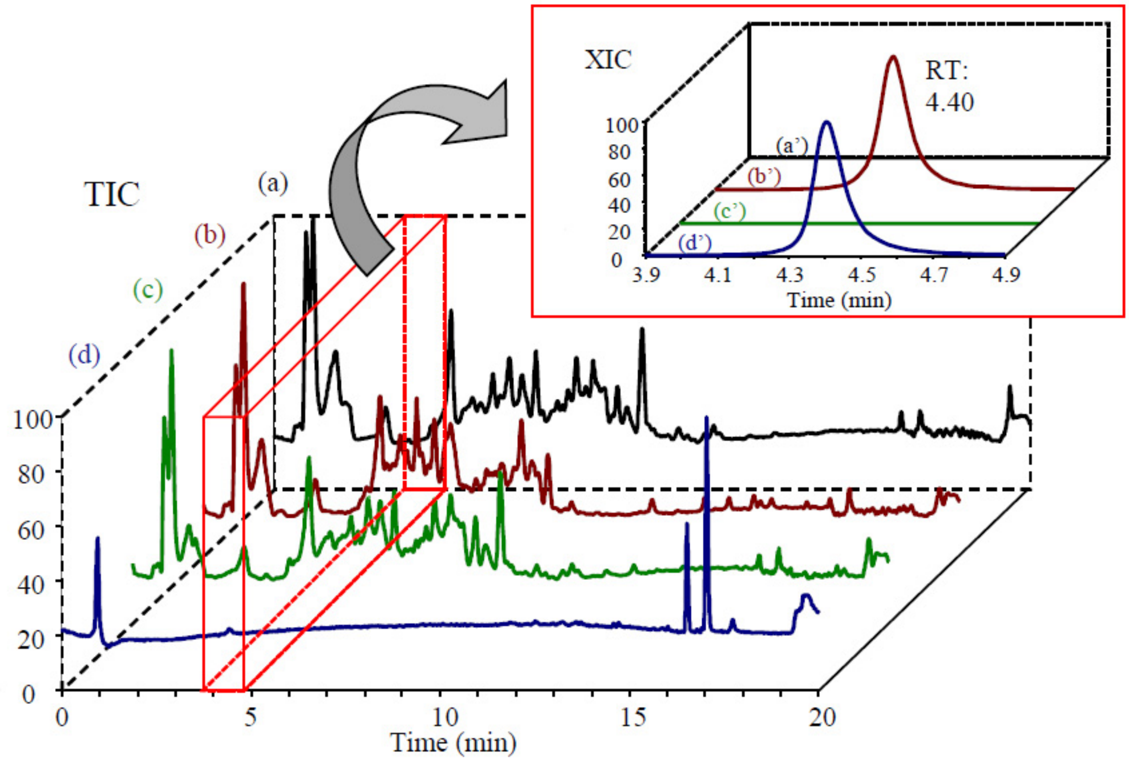

B

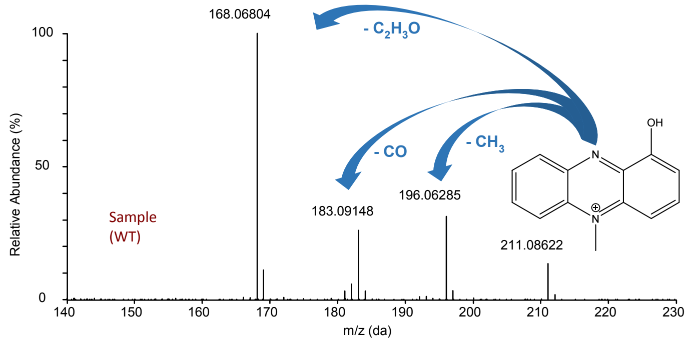

Supplementary Figure 5. UFLC-HRMS analysis of PAO1 secretants. (**A**) Total ion chromatograms (TIC) and extracted ion chromatograms (XIC) for *m/z* 211.08647 (tolerance; 5 ppm) of each sample: (a) LB medium; (b) WT secretant; (c) ΔlasR/rhlR secretant; (d) Pyocyanin (standard). (**B**) Product ion mass spectra for *m/z* 211.08622 (tolerance; 5 ppm) of WT sample in PRM mode.

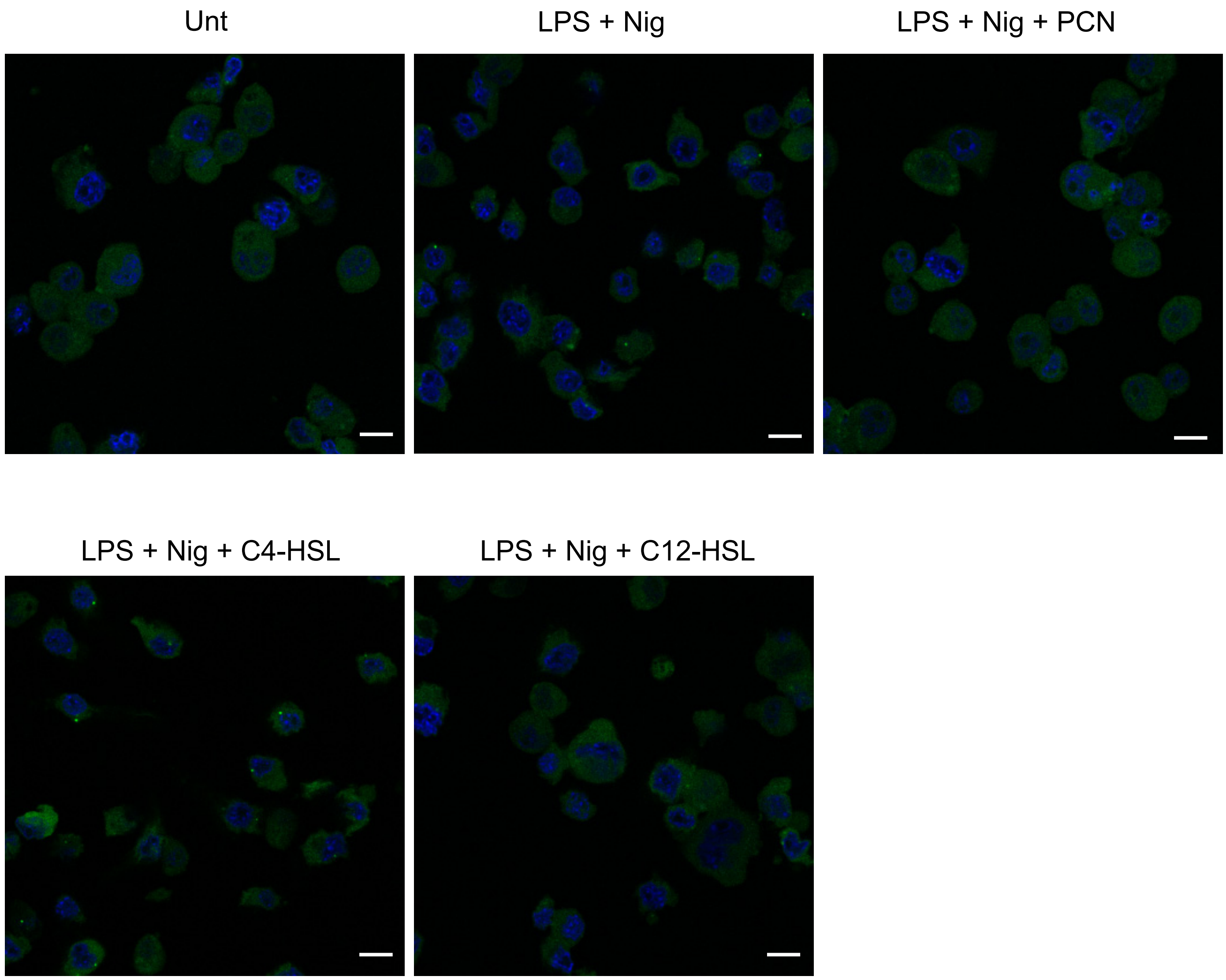

Supplementary Figure 6. Suppression of NLRP3 oligomerization by pyocyanin and 3-oxo-C12-HSL. NLRP3-GFP-expressing BMDMs were untreated or treated with LPS (0.25 μg/ml, 3 h) in the presence of 30 min pretreatment with pyocyanin (PCN, 50 μM) , C4-HSL (50 μM) or C12-HSL (50 μM), followed by the treatment with nigericin (Nig, 50 μM, 40 min). Cells were then observed by confocal microscope. Blue signals represent nuclear fluorescence. Blue signals represent nuclear fluorescence. Images were representative of three-independent samples. Scale bar, 10 μM
